# Anti-staphylococcus aureus adaptive immunity is impaired in end-stage renal disease patients on hemodialysis: one-year longitudinal study

**DOI:** 10.3389/fimmu.2023.1123160

**Published:** 2023-05-25

**Authors:** Anne Darbouret- Hervier, Nada Assi, Marie-Jeanne Asensio, Beatrice Bernabe, Aurélie Lechevallier, Raffaella Iantomasi, Bachra Rokbi, Elisabeth Botelho-Nevers, Sophie Ruiz

**Affiliations:** ^1^ Research Department, Sanofi, Marcy l’Etoile, France; ^2^ Infectious Diseases Department, University Hospital, Saint-Etienne, France; ^3^ CIC Inserm, University Hospital, Saint-Etienne, France; ^4^ CIRI – Centre International de Recherche en Infectiologie, Team GIMAP, University, Lyon, Université Jean Monnet, Inserm, CNRS, Saint-Etienne, France

**Keywords:** B-Lymphopenia, ESRD, hemodialysis, immune dysregulation, longitudinal study, *Staphylococcus aureus*

## Abstract

**Introduction:**

Patients with end-stage renal disease (ESRD) display defects in adaptive and innate immunity, increasing susceptibility to infection. *Staphylococcus aureus* (*S. aureus*) is a major cause of bacteraemia in this population and is associated with increased mortality. More information on the immune response to *S. aureus* in these patients is needed to inform effective vaccine development.

**Methods:**

A longitudinal prospective study was carried out at two medical centers and included 48 ESRD patients who started chronic hemodialysis (HD) treatment ≤3 months before inclusion. Control samples were taken from 62 consenting healthy blood donors. Blood samples were obtained from ESRD patients at each visit, on month (M) 0 (beginning of HD), M6 and M12. Around 50 immunological markers of adaptive and innate immunity were assessed to compare immune responses to *S. aureus* in ESRD patients versus controls to document the changes on their immune profile during HD.

**Results:**

*S. aureus* survival in whole blood was significantly higher in ESRD patients than in controls at M0 (*P*=0.049), while impaired oxidative burst activity was observed in ESRD patients at all timepoints (*P*<0.001). *S. aureus*-specific immunoglobulin G (IgG) responses to iron surface determinant B (IsdB) and *S. aureus* α hemolysin (Hla) antigens were lower in ESRD patients than in healthy donors at M0 (*P*=0.003 and *P*=0.007, respectively) and M6 (*P*=0.05 and *P*=0.03, respectively), but were restored to control levels at M12. Moreover, *S. aureus*-specific T-helper cell responses were comparable to controls for IsdB but were impaired for Hla antigen at all timepoints: 10% of ESRD patients responded to Hla at M0, increasing to 30% at M12, compared with 45% of healthy donors. B-cell and T-cell concentrations in blood were significantly reduced (by 60% and 40%, respectively) compared with healthy controls. Finally, upregulation of Human Leucocyte Antigen-DR (HLA-DR) and C-C chemokine Receptor type 2 (CCR2) was impaired at M0 but was restored during the first year of HD.

**Conclusion:**

All together, these results show that adaptive immunity was largely impaired in ESRD patients, whereas innate immunity was less impacted and tended to be restored by HD.

## Introduction

Chronic kidney disease (CKD) is a major public health concern, and patients with end-stage renal disease (ESRD) who require dialysis initiation or kidney transplantation are estimated at between 4 and 7 million worldwide (1). ESRD patients are immunocompromised and display significant immunological defects in both adaptive and innate immunity. These immunological defects are the result of the dysregulation of uremic metabolism and are comparable to premature aging of the immune system (2, 3). Although the exact mechanisms underlying these defects are not yet fully understood, they often result in an increased concentration of polymorphonuclear cells (PMN), a decreased concentration and functionality of whole lymphocytes, chronic activation of innate immune cells, and increased susceptibility to immune cell apoptosis.

Immune dysregulation in ESRD patients is also associated with low seroconversion rates and a rapid drop in antibody titers following vaccination (4, 5), potentially reducing the ability of these patients to respond to vaccines. This immune defect requires an adapted vaccination regimen, higher dose and/or increased injection frequencies (6–8). Immune dysregulation also makes ESRD patients susceptible to infections (9). Bacterial infections are one of the major causes of hospitalization and death in ESRD hemodialysis (HD) patients. *Staphylococcus aureus* (*S. aureus*) is one of the main pathogens responsible for bacteremia in this population (10). The high incidence of *S. aureus* infection in ESRD patients is exacerbated by recurrent exposure to the hospital environment and dependence on vascular access catheters. The incidence is approximately 40 cases per 1000 person-years, far higher than in the general population (<1 case per 1000 person-years) (11) and associated with a nine-fold increased risk of death (12). Methicillin-resistant *S. aureus* (MRSA) is responsible for a significant number of these deaths. Recurrent *S. aureus* infections and biofilm formation can cause long-lasting infections in ESRD HD patients, which are difficult to eradicate with antibiotic treatment. It is now accepted that humoral response alone is not sufficient to protect efficiently against *S. aureus* infection; specific T-cell response is also required. Several studies in murine models of infection or in patients with altered T-helper (Th) cell responses have demonstrated the requirement of Th1 and Th17 cells to sustain both the antibody response and neutrophil priming through cytokine production (13–16). ESRD patients must also display functional antigen presenting cells (APC) capable of sensing bacteria, and functional PMN, which are the main effectors required to eradicate the bacteria (17, 18).

Accordingly, the high frequency of infections in ESRD HD patients makes sense to use two *S. aureus* proteins as model antigens to assess the changes in their immune profile over the first year of HD. To evaluate the potential immunity against *S. aureus*, we designed a prospective study including ESRD patients who recently started HD treatment and followed the immune profile of these patients over their first year of HD. The start of HD treatment is a period during which patients are particularly susceptible to *S. aureus* infections. We simultaneously investigated around 50 immunological markers for each patient and processed all the data together to define the immunological status during the first year of HD with respect to the anti-*S. aureus* response.

## Materials and methods

### Study design

The study is a prospective, longitudinal study carried out at two centers, the University Hospital of Saint-Etienne and ARTIC 42, Saint Etienne, France. Consecutive ESRD patients, who started chronic HD treatment with a central venous catheter less than 3 months before inclusion, were invited to participate. Patients aged 18 years and above who accepted and provided written informed consent were included between January 2018 and December 2019. Exclusion criteria included positivity for human immunodeficiency virus, infection with hepatitis B or C viruses or anemia (hemoglobin <7 g/dL). Controls were healthy donors who volunteered at the French blood bank, Etablissement Français du Sang (EFS; Lyon, France), and provided informed consent for the use of their blood for scientific research purposes.

The conduct of this study was consistent with standards established by the Declaration of Helsinki and compliant with the International Conference on Harmonisation guidelines for Good Clinical Practice, including all local and/or national regulations and directives. The study was approved by the Review Board of Comité De Protection Des Personnes Est-III, CHRU Nancy, France, N°ID RCB: 2017-A00707646, N°CPP: 17.05.17.

### Data and sample collection

Deaths and *S. aureus* infections were recorded during the one-year follow-up period. Patient monitoring included three visits: at inclusion (ESRD M0), after 6 months (ESRD M6) and after 12 months (ESRD M12). At each visit and before each dialysis session, patients were tested for *S. aureus* carriage using nasal swabs. Additionally, 50 mL of blood was collected through the vascular access into lithium heparin tubes (BD Vacutainer, BD Biosciences) for subsequent biochemical and immunological analyses. Blood samples from controls were collected by venipuncture into citrate tubes at EFS. Whole leukocytes were isolated for immediate use and peripheral blood mononuclear cells (PBMCs) were isolated for immediate use or were frozen in liquid nitrogen for subsequent analysis. Plasma samples were also collected and stored at −20°C for subsequent analyses.

### Leukocyte and PBMC isolation

Leukocytes were isolated from 10 mL of blood following a 10-minute incubation with 90mL ammonium chloride-based lysing buffer (150 mM NH_4_Cl, 10 mM KHCO_3_, 1 mM EDTA, pH 7.4). Leukocytes were washed in RPMI 1640-hepes medium supplemented with 2 mM L-glutamine (*In vivo*Gen), 100 U/mL penicillin, 100 µg/mL streptomycin (Sigma-Aldrich) (RPMIc) and 0.5% bovine serum albumin (BSA) (Eurobio). Total leukocyte and PMN (defined as large leukocyte) concentrations were determined using a Multisizer cell counter (Beckman Coulter). For PBMC separation, blood was diluted with an equal volume of 0.9% NaCl (Aguettant) and `PBMCs were isolated by density gradient centrifugation, using ficoll-hypaque (Sigma-Aldrich) and leucosep tubes (Greiner). PBMCs were then washed in RPMIc plus 5% fetal bovine serum (FBS; Hyclone) and viable PBMCs were counted using viacount staining and a Muse cell counter (Millipore). An aliquot of the isolated PBMCs was cryopreserved in FBS 10% DMSO for subsequent ELISPOT interferon (IFN)-γ assay.

### *In vitro* activation of leukocytes and PBMCs with toll-like receptor agonists

Freshly purified PBMCs and leukocytes were plated in duplicate in RPMIc 5% FCS at 0.5 x 10^6^/well in a 96-well plate. Either 0.5 µg/mL E6020 TLR4-agonist (EISAI) or 2.2 µg/mL CpG TLR9-agonist (Avecia) was added. Leukocytes and PBMCs cultured in medium alone were used as negative controls of stimulation. Cells were incubated overnight at 37°C in a 5% CO_2_-humidified environment. Culture supernatants of PBMCs were then collected for evaluation of secreted inflammatory cytokines and leukocytes were stained for analysis by flow cytometry.

### Bacteria survival in whole blood

Blood was collected as described above and a 3 mL sample was mixed with 35 μL of newly thawed *S. aureus* strain Newman at ∼65 × 10^6^ CFU/ml. The blood/bacteria mixture was then incubated for one hour at 37°C under horizontal stirring. Bacterial viability was evaluated in the initial mixture and after the one-hour incubation in serial dilutions of blood/bacteria mixture on Trypticase Soy Agar (TSA; Biomerieux) plates followed by overnight incubation at 37°C. The percentage of bacterial survival after one hour-incubation was determined relative to the initial inoculum.

### Oxidative burst activity in neutrophils

Oxidative burst induced in PMNs was evaluated using flow cytometry, by measuring released H_2_O_2_ with dihydrorhodamine (DHR) 123 (Invitrogen). When oxidized by H_2_O_2_, DHR 123 is converted to fluorescent DHR^+^ which is excitable at 488 nm on the cytometer. The well-known S. *aureus* strain Newman, isolated from an osteomyelitis human infection (ATCC), was used for the assay. Bacteria were thawed on the day of the assay, washed in PBS and the concentration adjusted to 10^8^ CFU/mL in RPMIc 0.5% BSA. After leukocyte isolation, the number of PMNs among whole leukocyte population was estimated by counting the large cells on the Multisizer counter (Beckman coulter, 4.0). Native or heat-inactivated (56°C for 30 minutes) sera from ESRD patients and healthy donors were assessed in parallel. In a 96-deep well plate, the reagents were added in the following order: bacteria with sera, PMN, and DHR 123. 500 µL of RPMIc plus 0.5% BSA, containing 25 x 10^6^ bacteria, 0.25 x 10^6^ PMNs, 5µL of native or heat inactivated serum and 1 µg/mL of DHR 123, was incubated at 37°C with gentle agitation for 30 minutes. The oxidative burst reaction was stopped by placing the plate on ice. Data acquisition was performed with the Fortessa X20 flow cytometer using DIVA software (BD) and analysis was performed using Flow Jo software (BD). PMNs were identified on a side scatter/forward scatter (SSC/FSC) dot plot as large granular cells and results were expressed as the percentage of DHR-positive cells within the PMN population.

### Serum immunoglobulin titers

Anti-*S. aureus* specific IgG titers were evaluated by enzyme-linked immunosorbent assay (ELISA). Briefly, 96-well ELISA microplates were coated with either purified iron surface determinant B (IsdB) or *S. aureus* α hemolysin (Hla) recombinant antigens at 1µg/mL in 100mM carbonate buffer pH 9.6 (Sigma-Aldrich). IsdB and Hla recombinant antigens were both produced in *BL21 E. coli strain* (Invitrogen) and purified in-house. The antigens displayed a high degree of purity with low residual endotoxin levels (<5 EU/mg). Coated ELISA plates were incubated overnight at 4°C and then incubated with PBS plus 1% BSA as blocking buffer for one hour at 37°C. After washing, serum samples and reference serum were added in serial dilutions in PBS 0.05% Tween containing 1% BSA, and plates were incubated at 37 °C for additional 2 hours. After washing, 100 µL of peroxidase-conjugated goat anti-human IgG (Sigma-Aldrich) was added to the wells at the dilution of 1/2000. Tetramethylbenzidine substrate (Tebu-Bio) was then added to each well. The reaction was stopped using 1N HCl and absorbance was measured at 450 nm using Versamax (Molecular Devices).

Total serum IgG, IgA and IgM titrations were performed using Pentra C400 equipment (Horiba) and supplier kits and according to supplier protocol.

### IFNγ -producing cell ELISPOT

Nitrocellulose plates (Millipore) were coated with anti-human IFN-γ monoclonal antibody (mAb) (Mabtech), at a concentration of 15 µg/mL and incubated overnight at +4°C. Plates were then washed twice and blocked with AIMV medium (Invitrogen). PBMCs were thawed in FBS and washed twice in RPMI 10% FBS. The concentration of viable cells was adjusted to 2 x 10^6^ cells/mL in AIMV. One hundred µL of cell suspension was added per well followed by 100 µL per well of Hla or IsdB *S. aureus* antigen solution in duplicates, at a final concentration of 10 µg/mL for cell-specific activation. CPI (Immunospot) was used at 5 µg/mL as a positive control. Plates were incubated for 24 hours at 37°C and then washed. BAM-conjugated anti-IFN-γ mAb (Mabtech) was added and plates were incubated for 2 hours at room temperature. After washing, anti-BAM 490 (Mabtech) was added and incubated for 1 hour at room temperature. The plates were dried and spot counting was performed using Iris fluorospot reader (Mabtech). The number of IFN-γ-producing cells in the control medium was subtracted from the number of IFN-γ producing cells in activated assays, and the results were expressed as spot number per 10^6^ PBMCs.

### Fluorescent monoclonal antibody cocktails

The phenotype of leukocyte subpopulations was evaluated in freshly isolated leukocytes. Surface phenotyping was performed using the following 9-color panel of mAb: BUV395-conjugated anti-cluster of differentiation (CD)45 mAb and APC-H7-conjugated anti-CD3 mAb were purchased from BD Biosciences; BV421-conjugated anti-CD14 mAb, BV605-conjugated anti-CD19 mAb, BV711-conjugated anti-CD56 mAb, fluorescein isothiocyanate (FITC)-conjugated anti-CD4 mAb, phycoerythrin (PE)-conjugated anti-CD66b mAb, allophycocyanin (APC)-conjugated anti-T cell receptor (TCR)γδ and the live/dead marker Zombie Aqua were purchased from Biolegend.

To evaluate the phenotype of monocytes and neutrophils, we used freshly isolated PBMCs and freshly isolated leukocytes, respectively. Both cell suspensions were incubated overnight at 37°C with or without E6020 TLR4-agonist or CpG TLR2-agonist. Surface phenotyping of monocytes was performed using the following 8-color panel of monoclonal antibodies: BUV395-conjugated anti-CD45 mAb and BV421-conjugated anti-CD282 mAb were purchased from BD Biosciences; APC-FIRE-conjugated anti-CD14 mAb, BV605-conjugated anti-C-C chemokine receptor 2 (CCR2) mAb, BV711-conjugated anti- HLA-DR mAb, AF488-conjugated anti-CD86 mAb, APC-conjugated anti-CD284 mAb and the live/dead marker Zombie Aqua were purchased from Biolegend.

Surface phenotyping of neutrophils was performed using the following 8-color panel of mAb: BUV395-conjugated anti-CD45 mAb and BV421-conjugated anti-CD66b mAb were purchased from BD Biosciences; APC-FIRE-conjugated anti-CD11b mAb, BV605-conjugated anti-CD64 mAb, BV711-conjugated anti-CD16 mAb, FITC-conjugated anti-CD35 mAb, PE-conjugated anti-CD181 mAb and the live/dead marker Zombie Aqua were purchased from Biolegend. All fluorescent antibodies were previously titrated to determine their optimal concentration and fluorescence-minus-one controls were included.

### Immunostaining and flow cytometry analysis

For immunostaining, purified PBMCs or leukocytes were seeded in a 96-V-well plate at a concentration of 500,000 cells/well and cells were then washed in PBS 0.1% BSA (washing buffer). Fc receptors were blocked for 10 minutes with TruStain (BioLegend) diluted to 1/100 in washing buffer. The fluorescent antibody cocktails were added and incubated for 30 minutes. After washing, live/dead cells were identified by adding Zombie aqua for 15 minutes. After 2 additional washes, cells were immediately counted on the BD Fortessa X20 cytometer, using DIVA software (BD Biosciences). Compensations were automatically done by DIVA software with single fluorescent staining. To ensure consistent and reproducible Mean Fluorescent Intensities (MFI) over time, the voltages of each PMT were saved in the “application settings” of the instrument and the cytometer was calibrated daily with CST beads (BD Biosciences). Ten thousand events were acquired for each assay.

Analysis was performed using Flow Jo software (BD Biosciences) according to the following strategy. First, the cells acquired out of a stable flow were excluded using the dot plot FSC/Time. Second, doublets of cells were excluded using the dot plot FSC-Area/FSC-Height. Then, cellular debris and residual red cells were excluded from leukocytes using forward scatter (FSC: cell size) and side scatter (SSC: cell complexity). To determine leukocyte subpopulations, we first used a dot plot CD66b/CD14 to define the CD66^+^ PMN population, CD14^+^ monocyte population and CD66^-^ CD14^-^ lymphocyte population. Then, using a CD3/CD19 dot plot defined on the lymphocyte gate, we evaluated T- and B- cell populations respectively. In parallel using a CD3/CD56 dot plot defined on the lymphocyte gate, we evaluated natural killer (NK) cell and natural killer T (NKT) cell populations. Finally, using a CD4/TCRγδ dot plot defined on the CD3^+^ cell gate, we evaluated CD4^+^, TCRγδ^+^ and CD4^-^ cell populations. CD4^-^/TCRγδ^-^ cells were used to estimate CD8^+^ T cell numbers and CD3^+^ CD56^+^ cells were used to estimate NKT cell numbers.

Each leukocyte subpopulation was expressed as a percentage of total viable leukocytes. To determine the absolute number of each cell subpopulation per mL of blood, the percentage of each subpopulation was multiplied by the concentration of total leukocytes determined with Muse^®^ Cell Analyzer. The expression of various PMN and monocytes markers were measured respectively on CD66b^+^ and CD14^+^ cells and expressed as MFI.

### Statistical analyses

Descriptive and univariate analyses were performed using SAS statistical software (v9.4 on WISE environment) and multivariate analyses were conducted in RStudio using R statistical software (v.3.6). Variables were log_10_ transformed prior to statistical analysis. Distributions were examined through QQ-plots and Shapiro Wilks testing to identify those normally distributed and those that did not have a Gaussian distribution.

#### Correlation analysis

Spearman correlations were computed separately for each group (Controls, ESRD M0, ESRD M6, ESRD M12) given that most biological parameters did not have a Gaussian distribution. The correlation coefficient (ρ) was computed between all available biomarkers and between age (**Table S1**).

#### Univariate analyses

For each biological parameter, each of the sampling time-points (ESRD M0, ESRD M6, ESRD M12) was compared with the control group using a one-way parametric analysis of variance (ANOVA) model when the parameter displayed a normal distribution (with a repeated statement if needed to account for group heterogeneity). Otherwise a non-parametric Wilcoxon rank-sum test was applied. A Dunnett adjustment was applied to account for multiple testing in the parametric analysis and a false discovery rate (FDR) correction was applied in the non-parametric analysis. For pairwise comparisons between each sampling time-point, a Tukey adjustment was used in parametric analyses and an FDR correction in the non-parametric analyses. Further details of the methodologies for burst analysis and longitudinal analysis is available in **Tables S2**, **S3**.

#### Multivariate analyses

Sparse partial least squares discriminant analysis (sPLS-DA) was performed to identify linear combinations of 50 measured biological parameters that discriminate between two groups with above or below a defined burst value threshold: burst value threshold ≥25th percentile of control values or burst value <25th percentile of control values. This methodology was previously described in detail (19–21).

#### Mortality analysis

The association between the levels of three outlined lymphocyte variables (including B-lymphocytes, T-lymphocytes and TCRγδ T-cells) and the risk of death was investigated by using Cox proportional hazards regression models to estimate hazard ratios (HR) and 95% confidence intervals (95% CI). Breslow’s method was adopted for handling time ties computed to relate to the risk of death expressed as HR. The analysis was performed on the ESRD patients, using the latest values available for each subject. The time of exit was the date of death, loss-to-follow up or last sampling date. The analysis was adjusted for both patient comorbidity (Charlson index) and age.

## Results

### ESRD patient cohort

Forty-eight ESRD patients displaying similar stages of renal failure were included in the study. The cohort comprised 28 men and 20 women with a median age of 66 years, ranging from 24 to 82 years (**Table 1A**). The incidence of mortality was 13% and there were six deaths reported during the one-year follow-up: three before M6 sampling and three before M12 sampling. Five patients discontinued the study to receive dialysis elsewhere (two before M6 sampling and three before M12 sampling). Thus, 43 patients were studied at M6 and 37 at M12. Hemodialysis was performed on catheter in 38 patients (79.2%) at M0, in 22 patients (51.2%) at M6 and 18 (48.6%) at M12. Arterio-venous fistula replaced catheter progressively along the follow-up. The incidence of *S. aureus* infection was 6% during the follow-up year. Three out of 48 patients were infected, of which two were infected once and one was infected twice. Indeed, the progressive replacement of catheters by fistulas over time in ESRD cohort must have decrease the exposition to *S. aureus* and the risk of bacteremia. As expected, uremia and glycemia levels were elevated above normal values in this cohort (**Table 1B**). These values remained stable throughout the one-year follow-up. The complement factors evaluated (CH50, C3) were normal at inclusion and stable throughout the year of follow-up.

**Table 1A:** Patient characteristics – ESRD patient cohort.

Patient demographics	
Sex	
Men, n	28
Women, n	20
Median age (range), years	66 (24-82)
Median dialysis vintage (range), days	60 (25-120)
Incidence of diabetes	38%
Incidence of high blood pressure	35%
Median Charlson comorbidity index	3
*S. aureus* infected patients*, percent (n/M)	6% (3/48)
Deaths*, percent (n/M)	13% (6/48)

*S. aureus infections 1 bacteremia, 1 surgical site infection and 1 cutaneous infection (blood culture) and deaths are reported over the 12-month follow-up period.

**Table 1B:** Biochemical data and *S. aureus* carriage at each visit.

	M0 (N=48)	M6 (N=43)	M12 (N=38)
*S. aureus* carriage* (%)	26	32	21
Uremia (mmol/L)	22	23	24
Glycemia (mmol/L)	9	8	7
CH50 (units/mL)	71	73	77
C3 (g/L)	1.02	1.00	1.12

*S. aureus carriage determined by nasal swab. M, month.

Sixty-two healthy subjects from EFS were included as the control population. The ages of the control population ranged from 18 to 70 years old. Spearman correlations between age and each immune parameter were computed to exclude any confounding effect of age on the immune status of ESRD patients (ρ<0.5; **Table S1**). Moreover, where relevant, all statistical analyses were adjusted for age.

### Impairment of functional immunity against *S. aureus* was attributable to impaired serum but not to PMN in ESRD patients

**Figure 1:**
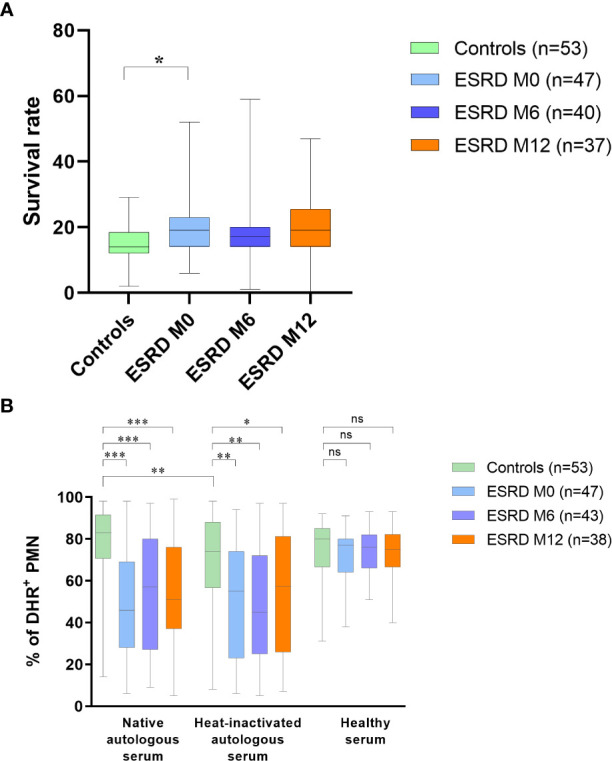
Bacteria survival in blood and oxidative burst measurement in neutrophils **(A)** Frequency of bacteria surviving in blood after one-hour incubation. **(B)** Frequency of activated polymorphonuclear cells in presence of *S. aureus* autologous or healthy serum. * p<0.05; ** p<0.01; *** p<0.001; ns, non significant. DHR, dihydrorhodamine; ESRD, end-stage renal disease; M, month; PMN, polymorphonuclear cells. The horizontal lines of the box plots indicate the first quartile (bottom line), the median (middle line) and the third quartile (top line); minimum and maximum values are indicated by the bottom and top of the vertical lines (whiskers).

The effect of immune dysregulation on effector immunity against *S. aureus* in ESRD patients was assessed by measuring the ability of the *S. aureus* Newman strain to escape bacterial clearance in peripheral blood over a 1-hour incubation period. Compared with healthy donors, *S. aureus* survival was significantly increased (p =0.049) in the whole blood of ESRD patients collected at patient inclusion (M0). However, the increase appeared to be transient and was no longer significant after 6 months or 12 months of HD (**Figure 1A**).

To further investigate functional immunity against *S. aureus* in ESRD HD patients, the oxidative burst activity of PMN against bacteria was assessed, using native or heat-inactivated autologous serum (**Figure 1B**). When PMN and *S. aureus* bacteria were co-incubated with autologous native serum, a significant decrease in oxidative burst activity (p <0.01; **Table S2**) and a higher heterogeneity of response was measured in the ESRD cohort compared with the controls. These impairments persisted throughout the one-year follow-up period in the ESRD cohort. Compared with native sera, heat inactivation, which is deleterious for complement activity, induced a significant decrease in burst activity in the control group (p=0.001; **Table S3**) but did not significantly affect burst activity in the ESRD cohort.

When PMN and *S. aureus* were co-incubated with healthy serum instead of autologous serum, oxidative burst activity was restored in ESRD HD patients (**Figure 1B**). Burst impairment in the ESRD HD cohort may therefore be attributed to a defect in their serum compounds rather than to a defect in their neutrophils.

### Anti-*S. aureus* humoral immunity is impaired in ESRD HD patients

**Figure 2:**
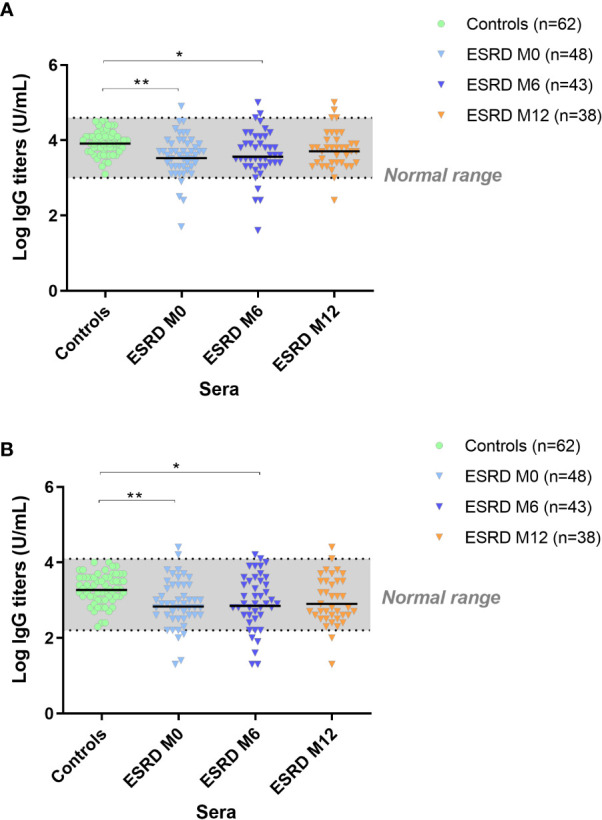
IgG titers anti-IsdB **(A)**. IgG titers anti-Hla **(B)**. *S. aureus* antigen-specific IgG response. *p<0.05; **p<0.01. ESRD, end-stage renal disease; Hla, α hemolysin; IgG, immunoglobulin G; IsdB, iron surface determinant B; M, month. The horizontal line indicates geometric means.

Specific IgG responses were assessed by ELISA on two vaccine prototype antigens: IsdB, a membrane antigen and Hla, a secreted antigen. In ESRD HD patients, anti-IsdB IgG ELISA titers were lower at M0 (p=0.003) and M6 (p=0.05) compared with control subjects. However, at M12, anti-IsdB IgG titers in ESRD patients were similar to those of the controls (**Figure 2A**). Similarly, anti-*S*. *aureus* Hla IgG titers were significantly lower in ESRD HD patients at M0 (p=0.007) and M6 (p=0.03) compared with the control group but were not different at M12 (**Figure 2B**), indicating that the anti*-S. aureus*-specific response was restored at the end of the one-year follow-up.

### Anti-*S. aureus* Th-cell immunity is impaired in ESRD patients

**Figure 3:**
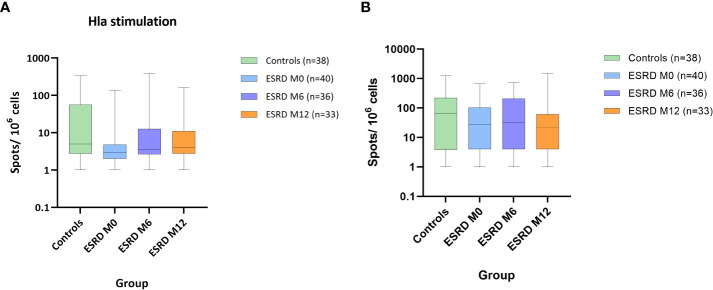
*S. aureus* antigen-specific IFNγ response. ESRD, end-stage renal disease; Hla, α hemolysin; IFNγ, interferon γ; IsdB, iron surface determinant B; M, month. Hla stimulation **(A)**. IsdB stimulation **(B)**. Data show number of spots per 10^6^ PBMC. For each box and whisker plot, the horizontal lines of the box indicate the first quartile (bottom line), the median (middle line) and the third quartile (top line); minimum and maximum values are indicated by the bottom and top of the vertical lines (whiskers).

To complete the monitoring of the adaptive response, Th-cell responses specific to *S. aureus* IsdB and Hla antigens were assessed *ex vivo* in PBMC using T-cell ELISPOT. In each group, individuals displaying more than 10 spots per 10^6^ PBMC were identified as responders to *S. aureus* prototype antigens. Forty-five percent of healthy donors were responders to Hla. In comparison, only 10% of ESRD patients showed such a response at M0 (**Table 2**). Moreover, in ESRD responders, the frequencies of IFNγ-producing Th-cells specific to Hla tended to be lower at M0 than in healthy donors (**Figure 3A**). Over the one-year follow-up, the frequency of ESRD responders gradually increased to around 30% (**Table 2**). Unlike Hla, no differences between ESRD patients and healthy donors were highlighted for Th-cell responses specific to IsdB at M0, M6 or M12 (**Figure 3B**). Additionally, the level of specific Th-cell response in *S. aureus* infected ESRD patients during the study did not differ compared with non-infected patients (data not shown).

**Table 2:** Frequency of patients displaying a *S. aureus* antigen specific IFNγ response.

	EFS (n=62)	ESRD M0 (n=48)	ESRD M6 (n=43)	ESRD M12 (n=38)
Hla response	45%	10%	28%	30%
IsdB response	66%	65%	69%	67%

EFS, Etablissement Français du Sang; ESRD, end-stage renal disease; Hla, α hemolysin; IsdB, Iron surface determinant B; M, month.

The number of IFNγ secreting cells was evaluated by Fluorospot. PBMC were restimulated 24 hours in the presence of Hla or IsdB *S. aureus* antigens at 10µg/mL. IFNγ response was estimated to be positive above the threshold of 10 spots per million PBMC.

### B- and T-lymphocyte blood concentrations were dramatically decreased in ESRD-patients

**Figure 4:**
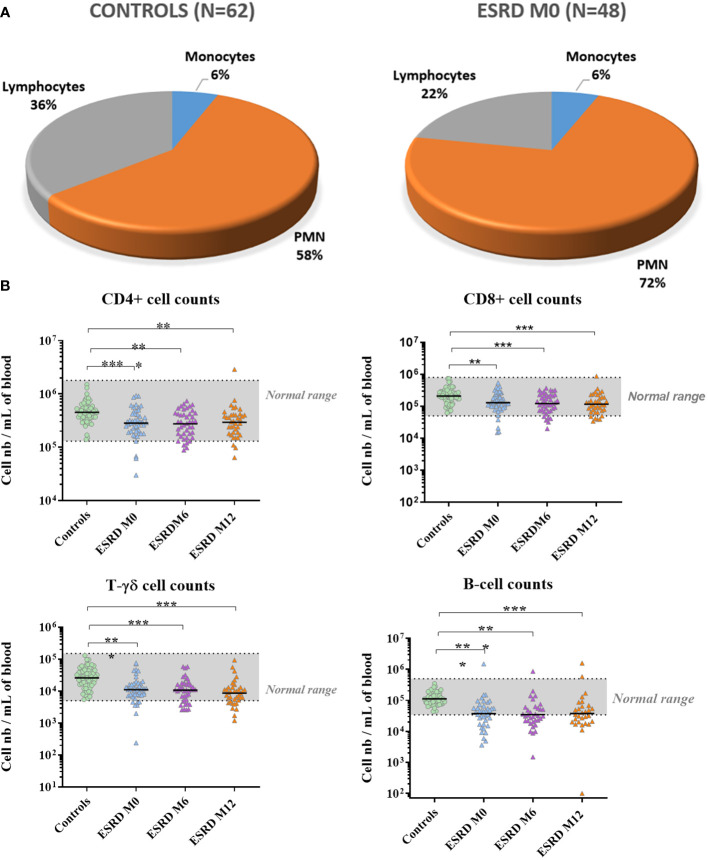
Lymphocyte numbers. **(A)** Relative numbers of polymorphonuclear cells, monocytes and lymphocytes. **(B)** Lymphocyte counts over the one-year follow-up period. *p<0.05; **p<0.01; ***p<0.001. CD, cluster of differentiation; ESRD, end-stage renal disease; M, month; N, number of subjects; PMN, polymorphonuclear cells. The horizontal line indicates geometric means.

The concentration of leukocyte subsets was assessed in peripheral blood collected at M0, M6 and M12. The whole lymphocyte population was largely decreased in ESRD patients at inclusion (M0) compared with healthy donors (p<0.01; S3), which persisted throughout the one-year clinical follow-up (**Table 3**). Simultaneously, an increase in neutrophils was observed in ESRD patients (p=0.006), leading to an increase in the myeloid to lymphoid cell ratio in this cohort (**Figure 4A**). Contrary to NK- and NKT-cell numbers, which were slightly reduced in ESRD patients, T- and B-cells were considerably reduced (**Figure 4B**). ESRD patients displayed a 60% reduction in B-cell concentration and a 40% reduction in T-cell concentration, compared with healthy controls. Further investigation of T-cell subsets showed that CD4^+^, CD8^+^ and TCRγδ^+^ T-cell populations were all dramatically reduced in ESRD patients (p<0.001; S3). TCRγδ^+^ T cells appeared to be the most impacted, with 17 out of 48 ESRD patients displaying a concentration below the normal range defined for the healthy population (**Figure 4B**).

**Table 3:** Absolute numbers of leukocyte subsets.

Cell subset	EFS controls (n=62)	ESRD patients at M0 (n=48)	ESRD patients at M6 (n=43)	ESRD patients at M12 (n=38)
Leukocytes (10^6^/mL)	3.23 (2.98, 3.51)	3.65 (3.15, 4.22)	3.17 (2.87, 3.50)	3.51 (2.96, 4.16)
Monocytes (10^6^/mL)	0.18 (0.16, 0.20)	0.20 (0.17, 0.24)	0.18 (0.16, 0.21)	0.18 (0.14, 0.22)
Neutrophils (10^6^/mL)	1.83 (1.65, 2.03)	2.55 (2.15, 3.04) **	2.17 (1.93, 2.45)	2.43 (1.99, 2.93)
Lymphocytes (10^6^/mL)	1.08 (0.99, 1.19)	0.69 (0.59, 0.81) ***	0.68 (0.58, 0.79) ***	0.72 (0.59, 0.88) **
T Lymphocytes (10^6^/mL)	0.73 (0.67, 0.81)	0.45 (0.37, 0.54) ***	0.43 (0.36, 0.51) ***	0.44 (0.36, 0.54) ***
B Lymphocytes (10^4^/mL)	11.39 (10.1, 12.8)	3.64 (2.67, 4.99) ***	3.83 (2.75, 5.35) ***	4.40 (2.94, 6.61) **
NK cells (10^4^/mL)	11.00 (9.35, 12.9)	7.13 (5.80, 8.74) *	8.48 (6.91, 10.4)	9.92 (7.64, 12.9)
NKT cells (10^4^/mL)	4.18 (3.48, 5.01)	2.80 (2.04, 3.83)	2.71 (1.98, 3.70)	2.64 (1.97, 3.54)
CD4+ T-lymphocytes (10^4^/mL)	45.42 (40.6, 50.8)	28.27 (23.4, 34.3) ***	27.56 (23.0, 32.8) ***	29.12 (23.4, 36.3) **
CD8^+^ T-lymphocytes (10^4^/mL)	20.95 (18.3, 24.0)	12.95 (10.4, 16.2) **	12.19 (9.80, 15.20) **	11.47 (9.14, 14.40) ***
TCRγδ T-lymphocytes (10^4^/mL)	2.60 (2.15, 3.13)	1.10 (0.82, 1.44) ***	1.06 (0.82, 1.36) ***	0.85 (0.64, 1.13) ***

CD, cluster of differentiation; EFS, Etablissement Français du Sang; ESRD, end-stage renal disease; NK, natural killer; TCR, T cell receptor.

Absolute numbers of leukocyte subpopulations, expressed as the number of cells per mL of blood: geometric means with geometric CI.

In bold: significant difference compared with control group: * p<0.05; ** p<0.01; ***p<0.001.

B cells were the most impacted by ESRD status. B-cell concentrations in ESRD patients at M0, M6 and M12 were significantly lower than those measured in healthy donors (p ≤ 0.001; **Table S2**). In addition, 28 out of 48 ESRD patients displayed B-cell concentrations below the normal range defined for the healthy controls. For the ESRD cohort, the geometric mean for B-cell concentration (3.6 x 10^4^ cells/mL of blood) was below the normal range.

**Figure 5:**
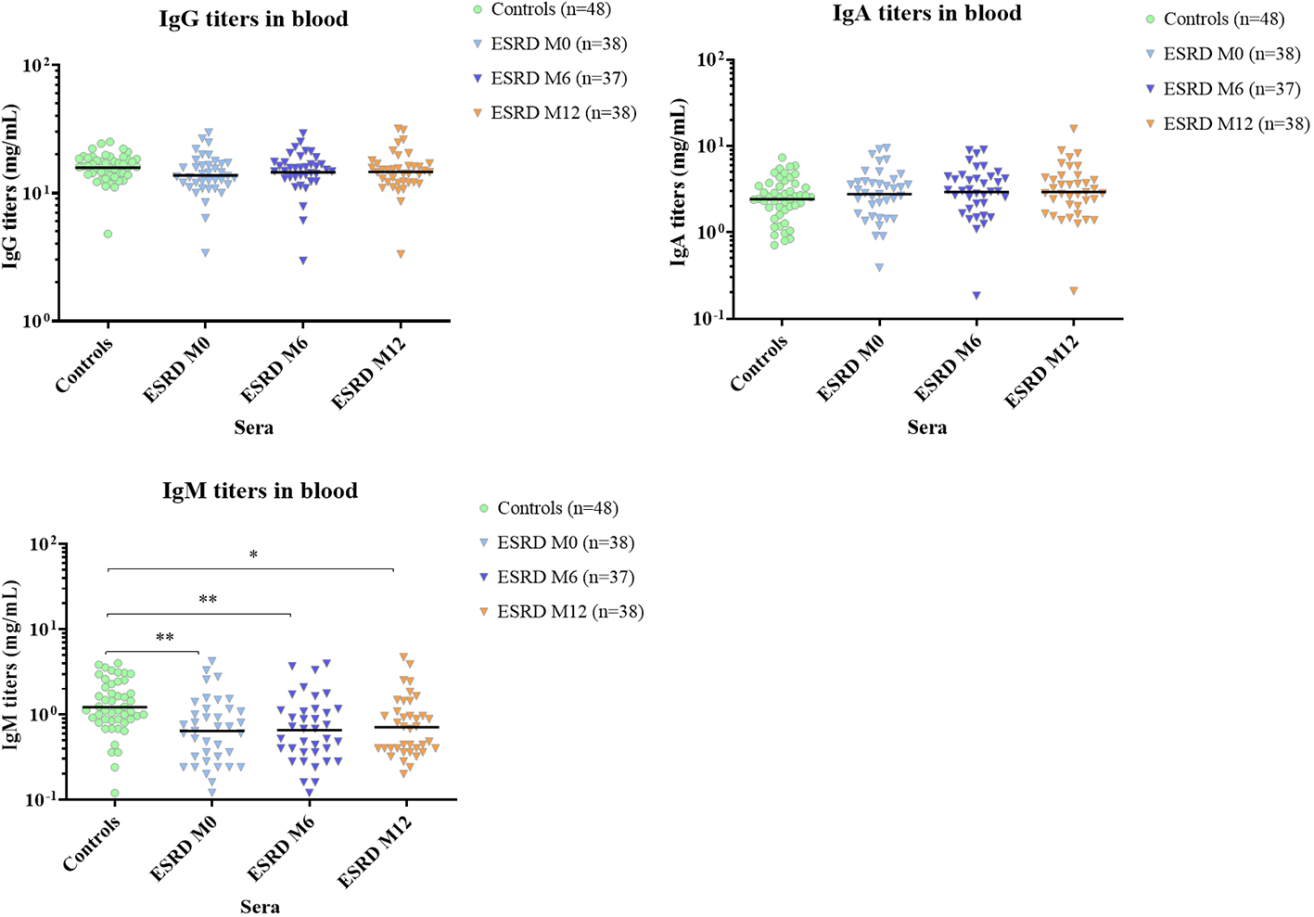
Total serum Ig titers. * p<0.05; ** p<0.01. ESRD, end-stage renal disease; Ig, immunoglobulin; M, month. The horizontal line indicates geometric means.

Concomitantly with the decrease in B cells, total serum IgM titers were considerably reduced at inclusion (p=0.004) and this decrease was maintained throughout the one-year follow-up (**Figure 5**). Conversely, total serum IgG and IgA titers were unaltered in ESRD patients compared with healthy donors.

### Potential association between B-cell concentration and ESRD patient outcome

Given that B cells, total T cells and TCRγδ^+^ T cells were decreased in ESRD patients, a possible correlation of these cell concentrations with ESRD patient outcome was further investigated. Over the one-year HD protocol, six male patients died before the end of the study. A further five patients were either transferred to another dialysis center or started peritoneal dialysis and were excluded from the analysis. Cox proportional hazards models showed that high B-cell depletion in ESRD patients tended to be associated with a higher risk of mortality (p=0.059; **Table 4**). Although total T cells and TCRγδ^+^ T cells were significantly depleted, no significant association with ESRD patient outcome was noted in this study.

**Table 4:** B-cell number was associated with patient outcome.

	Beta coefficient	HR (95% CI)	P value
B lymphocytes	- 1.69	0.18 (0.03, 1.07)	0.0589
T lymphocytes	-0.49	0.61 (0.004, 98.36)	0.850
TCRγδ lymphocytes	-0.42	0.66 (0.04, 10.11)	0.766
Age	0.14	1.15 (0.97, 1.36)	0.116
Comorbidity	-0.14	0.87 (0.54, 1.43)	0.588

CI, confidence intervals; HR, hazard ratio; TCR, T-cell receptor.

HR and their associated 95% CI are presented along with original linear estimates (beta coefficients) and p-values. These results stem from a series of multivariate Cox proportional hazard models examining the association between each of the presented variables and mortality. ESRD patient comorbidities were evaluated using Charlson comorbidity index.

### Monocyte impairments in ESRD patients were restored after one-year of hemodialysis

The concentration of the monocyte population was not impaired in ESRD patients compared with controls (**Table 5**). To further investigate monocyte functionality in ESRD patients, fresh PBMC were stimulated *in vitro* with either synthetic unmethylated CpG oligodeoxynucleotides, a TLR9 agonist, or with E6020, a TLR4 agonist. Monocyte functionality was assessed by flow cytometry, monitoring both basal and activated expressions of five key markers involved in inflammatory response induction (TLR2 and TLR4), monocyte migration (CCR2) and initiation of the adaptive immune response (HLA-DR and CD86).

**Table 5:** Phenotype of innate cells.

Cellular subset	Marker	Controls (n=58)	ESRD patients at M0 (n=46)	ESRD patients at M6 (n=42)	ESRD patients at M12 (n=38)
**Monocytes**	Basal CD86	1091 (1003,1187)	1019 (931, 1115)	1118 (1038, 1205)	1065 (1003, 1130)
	Activated CD86	919 (845, 999)	998 (906, 1101)	1062 (979, 1153)	1010 (962, 1060)
	Basal CCR2	2880 (2458, 3374)	2480 (2033, 3026)	3064 (2597, 3613)	2948 (2428, 3581)
	Activated CCR2	909 (839, 984)	675 (610, 747)***	757 (714, 803)**	821 (784, 861)
	Basal TLR2	5582 (5120, 6086)	4587 (4025, 5228)*	4510 (4091, 4972)*	4903 (4457, 5394)
	Activated TLR2	9792 9146, 10483)	8228 (7504, 9023)*	7833 (7181, 8545)**	8144 (7402, 8959)*
	Basal TLR4	2428 (2057, 2866)	2823 (2280, 3494)	1933 (1644, 2272)	1634 (1466, 1820)
	Activated TLR4	2367 (1989, 2816)	2666 (2184, 3255)	1873 (1593, 2202)	1589 (1417, 1782)
	Basal HLADR	3847 (3333, 4441)	2478 (2056, 2987)**	2998 (2604, 3452)	3895 (3283, 4622)
	Activated HLADR	12696 (10915, 14768)	6282 (5270, 7487)***	8799 (7505, 10317)*	11418 (9569, 13623)
	CCR2 ratio ^x^	3,17 (2.76, 3.64)	3,68 (3.04, 4.44)	4,05 (3.44, 4.76)	3,59 (2.99, 4.31)
	TLR2 ratio ^x^	0,57 (0.53, 0.61)	0,56 (0.51, 0.61)	0,58 (0.52, 0.63)	0,60 (0.54, 0.67)
	HLA-DR ratio ^x^	0,30 (0.27, 0.34)	0,39 (0.33, 0.47)*	0,34 (0.30, 0.39)	0,34 (0.29, 0.40)
**PMN**	Basal CD11b	1506 1286, 1763)	1450 (1278, 1644)	1413 (1211, 1649)	1266 (1066, 1503)
	Activated CD11b	2334 (2070,2631)	1804 (1608, 2025)*	1831 (1578,2125)	1599 (1387, 1844)*
	Basal CD35	1667 (1549, 1794)	1880 (1732, 2040)	1741 (1544, 1964)	1758 (1608, 1921)
	Activated CD35	2416 (2232, 2616)	2590 (2358, 2845)	2492 (2162, 2873)	2224 (1985, 2492)
	Basal CXCR1	5758 (5318, 6236)	5131 (4605, 5717)	5783 (5284, 6330)	5794 (5332, 6296)
	Activated CXCR1	2345 (2027, 2714)	2394 (2044, 2804)	2543 (2122, 3048)	2521 (2138, 2973)
	basal CD16	45352 (41191, 49933)	37751 (34648, 41132)*	38521 (35460, 41848)	38978 (35742, 42507)
	Activated CD16	32006 (29406, 34836)	28761 (26094, 31700)	28357 (26029, 30893)	27950 (25470, 30672)
	CD11b ratio ^x^	0,65 (0.58, 0.72)	0,80 (0.75, 0.87)*	0,77 (0.73, 0.82)	0,86 (0.80, 0.93)
	CD35 ratio ^x^	0,69 (0.64, 0.74)	0,73 (0.66, 0.80)	0,70 (0.64, 0.76)	0,80 (0.73, 0.87)
	CD16 ratio ^x^	1,42 (1.31, 1.54)	1,31 (1.22, 1.41)	1,36 (1.27, 1.45)	1,40 (1.28, 1.53)
**PBMC**	Secreted IL1β	1542 (1104, 2153)	874 (575, 1330)	1292 (944, 1767)	1305 (1025, 1662)
	Secreted IL10	571 (445, 734)	514 (374, 706)	560 (438, 715)	530 (419, 669)
	Secreted TNFα	20500 (14947, 28117)	18580 (13072, 26408)	29300 (22004, 39016)	23778 (18506, 30551)

CCR2, C-C chemokine receptor 2; CD, cluster of differentiation; CXCR1, C-X-C chemokine receptor 1; HLA-DR, human leucocyte antigen DR; IL, interleukin; M, month; PBMC, peripheral blood monocyte; PMN, polymorphonuclear cell; TLR, toll-like receptor; TNFα, tumor necrosis factor α; ^x^, ratio of basal mean fluorescent intensities/activated mean fluorescent intensities.

For PMNs and monocytes, marker expression levels were expressed as the Mean Fluorescence Intensity (MFI): geometric mean with geometric CI.

For PBMCs, cytokine concentration was expressed in pg/mL: geometric mean with geometric CI.

In bold: significant differences compared with control group: * p<0.05; **p<0.01; ***p<0.001.

TLR2 basal and activated expression levels on monocytes were significantly down-regulated in ESRD patients at M0 compared with healthy donors; this down-regulation persisted throughout the one-year follow-up (**Tables 5**; **S2**). However, the basal/activated expression ratio did not differ between ESRD patients and controls. In ESRD patients, basal and activated TLR4 expression levels on monocytes progressively decreased from M6 to M12 and were significantly downregulated at M12, compared with M0 (**Tables S4**, **S2**).

Basal expression of CCR2 on monocytes was not significantly modified in ESRD patients compared with controls. However, under TLR4-agonist activation, CCR2 was down-regulated to a greater extent in ESRD patients than in healthy controls at M0 (p ≤ 0.001). This difference in CCR2 down-regulation remained significant, albeit to a lesser extent, at M6 (p=0.04) and was no longer evident after 12 months of HD. In addition, longitudinal statistical analysis confirmed that the restoration of CCR2 activation during HD occurred at the level of each patient (p=0.006, M0 vs M12; **Table S5**).

HLA-DR basal and E6020-activated expression on monocytes was significantly decreased in ESRD patients compared with healthy donors at M0 (p ≤ 0.001). The significant decrease of HLA-DR-activated expression was still observed in ESRD patients at M6 (p=0.02). However, basal and activated expression of HLA-DR was gradually restored from M0 to M12 and was similar to the expression levels observed in healthy controls at the end of the one-year follow-up. Moreover, the basal/activated expression ratio was significantly different between ESRD patients and healthy controls at M0 (p<0.05; **Table S2** **)**, reflecting a decrease in monocyte activation. Unlike HLA-DR, CD86 was not altered in ESRD patients compared to the control group.

### Neutrophil phenotype was not altered in ESRD patients

Neutrophils are the main effector cells for *S. aureus* clearance. Neutrophil concentration was increased in ESRD patients at inclusion (M0) compared with healthy controls (p=0.006) and returned to a physiological level after 6 months of HD (**Table 5**). To further investigate neutrophil functionality in ESRD patients, whole leukocytes were stimulated *in vitro* and the expression of four key surface markers (C-X-C chemokine receptor 1 [CXCR1]. CD11b, CD16 and CD35) involved either in migration or opsonophagocytic activity was monitored using flow cytometry. No difference in CXCR1 expression was observed between the ESRD patients and controls. Similarly, no difference was observed in CD11b basal expression between ESRD patients and controls. However, upon activation with CpG, CD11b expression was significantly decreased in ESRD patients compared with the controls at M0 (p=0.02). This reduced expression in ESRD patients was greater at M12 (p=0.002). The basal to activated expression ratio was significantly different between ESRD patients and controls at M0 (p<0.01), reflecting a decrease in PMN activation.

The basal expression of CD16 on neutrophils was decreased in ESRD patients compared with healthy controls at M0 (p=0.02) but the decrease was not more significant at M6 and M12. The basal expression of CD35 as well as expression on activated neutrophils remained unchanged in ESRD patients compared with controls.

### Multivariate analysis revealed a major impairment of the humoral response in ESRD patients

**Figure 6:**
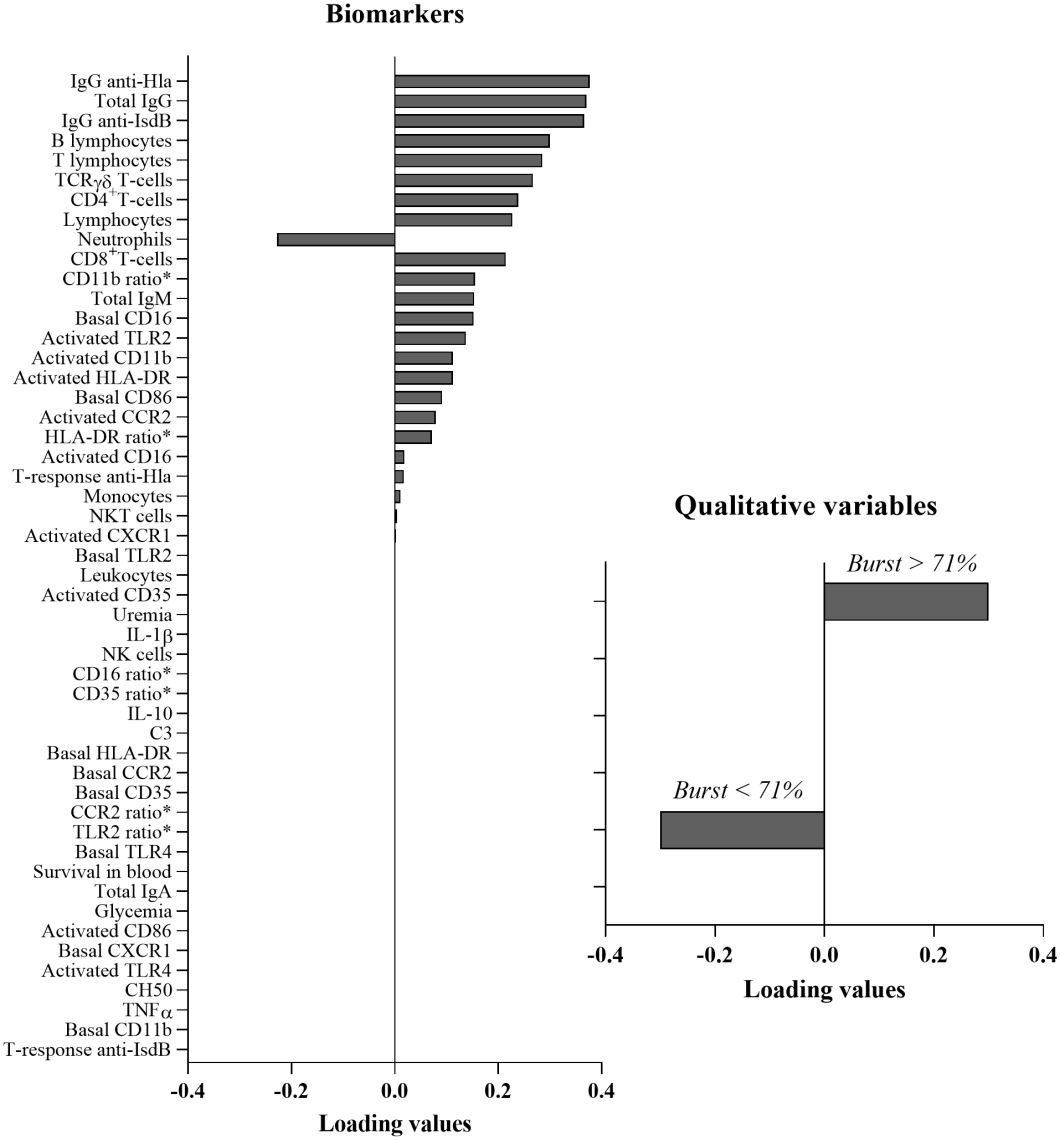
Multivariate analysis: Sparse partial least squares discriminant analysis. Positive loading values are associated with functional oxidative burst and negative loading values are associated with impaired oxidative burst. CCR2, C-C chemokine receptor 2; CD, cluster of differentiation; CXCR1, C-X-C chemokine receptor 1; Hla, α hemolysin; HLA-DR, Human leucocyte antigen DR; Ig, immunoglobulin; IL, interleukin; IsdB, iron surface determinant B; M, month; NK, natural killer; TCR, T cell receptor; TLR, toll-like receptor; TNFα, tumor necrosis factor α.*Ratio is the ratio of mean fluorescent intensity (basal marker)/mean fluorescent intensity (activated marker).

**Figure 7:**
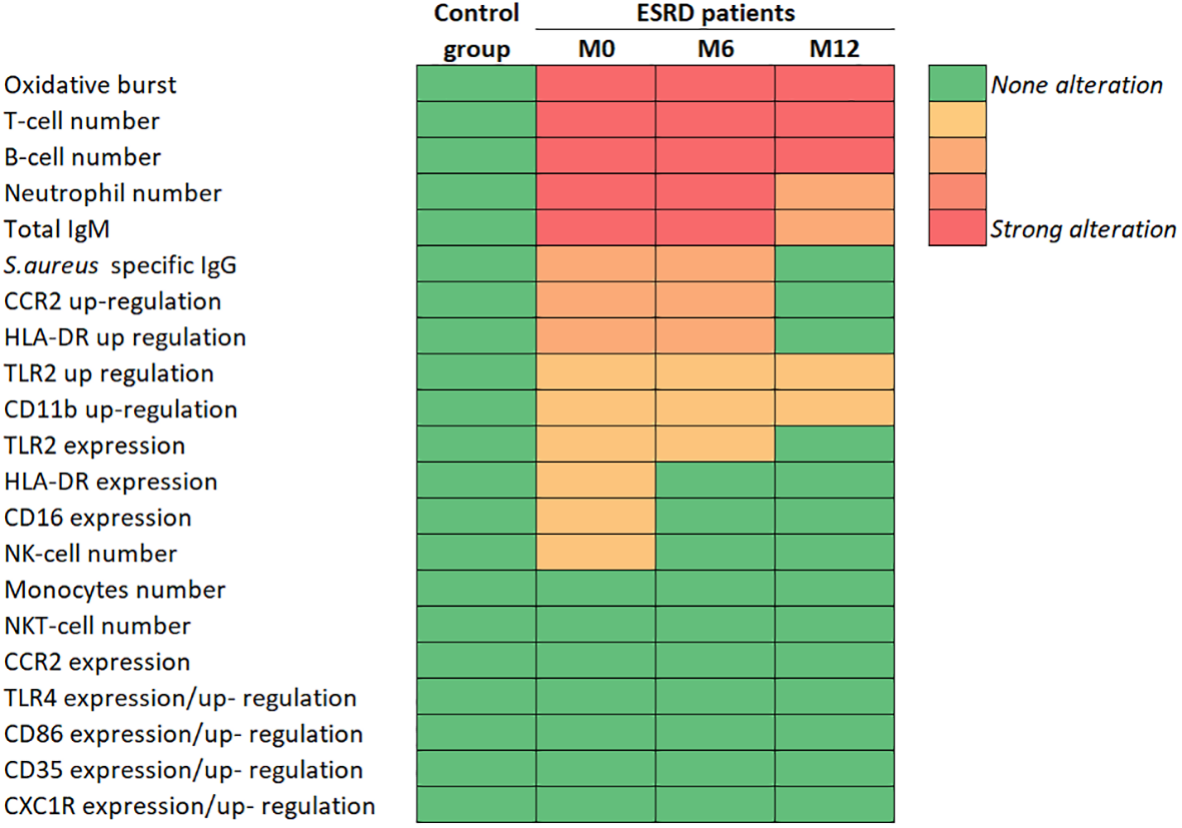
Summary of main immunological results. CCR2, C-C chemokine receptor 2; CD, cluster of differentiation; CXCR1, C-X-C chemokine receptor 1; ESRD, end-stage renal disease; HLA-DR, Human leucocyte antigen DR; Ig, immunoglobulin; IsdB, iron surface determinant B; M, month; NK, natural killer; TLR, toll-like receptor.

To identify an immunological signature that can discriminate between low and high oxidative burst levels, a sPLS-DA analysis was conducted including data from ESRD HD patients at inclusion (M0) and data from controls (**Figure 6**). Immunological signatures were identified mirroring a data-driven discrimination between individuals based on levels of oxidative burst activity. Individuals were classified into two groups according to their level of oxidative burst activity (expressed as a percentage of DHR^+^ PMN). The cut-off value to separate the two groups was 71% and was determined as the 25^th^ percentile of healthy individual distribution (**Figure 1A**, native serum). ESRD and healthy individuals displaying a percentage value above 71% of oxidative burst were considered as having functional oxidative burst activity, whereas those with a value below 71% were considered as having altered oxidative burst activity. Various immunological parameters could be associated with functional or impaired oxidative burst (**Figure 6**). The loading value of each biological parameter analyzed in sPLS-DA showed that humoral immunity (positive loadings) was predominantly associated with the oxidative burst functionality (displaying positive loadings as well) (**Figure 6**). IgG specific to *S. aureus*, total IgG and B-cell number displayed the highest loading values. TCRγδ+ and CD4+ T-cells displayed lower values but could also be associated with the functionality of oxidative burst. To a lesser extent, CD8^+^ T-cell numbers and PMN numbers also appeared to be associated with oxidative burst functionality: positively for CD8+ T-cell number and negatively for PMN number. A summary of immunological results can be seen in **Figure 7**.

## Discussion

In the present study, innate and natural anti-S. *aureus* immunity were evaluated within a cohort of 48 ESRD patients who recently started HD treatment and followed over one year-HD treatment. Our results do not anticipate the immune status of these patients beyond this timeframe. They may be in contradiction with previously published data concerning patients after several years of dialysis.

### Added value of the longitudinal study

The longitudinal design allowed us to monitor multiple parameters of innate and adaptive immunity in the same ESRD HD patient over a one-year follow-up period and assess the effect of HD on immune dysregulation and its impact on immune responses to *S*. *aureus*. Several impaired biomarkers were irreversibly dysregulated over this period, mainly those linked to adaptive immunity. The reduction in concentrations of all lymphocyte subpopulations was sustained throughout the study. Conversely, some impaired biomarkers had returned to their normal ranges by month 12. Indeed, the number of PMNs, which were increased at study inclusion, returned to the concentration measured in the control group after one year of HD. Similarly, CCR2 and HLA-DR expression, which were dysregulated on activated monocytes at study inclusion, returned to normal expression levels after one year of HD. These data suggest that ESRD HD immune system would be better armed, for antigen presentation and APC recruitment, after one year than at the start of HD. However, we cannot exclude that this beneficial effect is restricted to the first year of HD.

### Anti-*S. aureus* specific immunity

Opsonophagocytosis and oxidative burst activity are key immune mechanisms involved in bacterial clearance during *S. aureus* infection (22–24). In this longitudinal study, deficient oxidative burst activity was observed in PMNs from ESRD patients, co-incubated with *S. aureus* Newman bacteria, compared with those from controls, as previously reported (25, 26). Moreover, the oxidative burst impairment was sustained during the one-year ESRD HD patient follow-up period. Under the experimental conditions of this study, altered serum compounds, rather than neutrophil function failure, appeared to be responsible for the oxidative burst impairment. The significant decrease in *S. aureus*-specific opsonizing IgG could be one of the serum parameters contributing to the impaired oxidative burst activity in ESRD patients (25, 26).

In addition to opsonizing IgG decrease, neutralizing antibodies specific to Hla, one of the main *S. aureus* virulence factors, often targeted in anti-*S. aureus* therapies and vaccines (27), were also reduced in ESRD HD. These data inform on a global depletion of natural anti-*S*. *aureus* humoral immunity, which is likely linked to global B-cell depletion.

The survival of bacteria was also significantly increased in peripheral whole blood from ESRD HD patients compared with controls at the time of inclusion, suggesting that oxidative burst deficiency in ESRD HD patients increased bacterial resistance to clearance. Six months after patient inclusion, the increased survival of bacteria in blood from ESRD HD patients was no longer observed, although the oxidative burst activity remained altered until the end of the follow-up. One hypothesis to explain this discrepancy could be the occurrence of NETosis, an anti-infection mechanism deployed by PMNs, independent of opsonophagocytosis and independent of specific antibodies. HD treatment could favor NETosis by reducing uremic toxins in the blood. Moreover, NETosis has been shown to be effective against *S. aureus* infection (28) and to be increased in ESRD patients (29).

Several studies in mice and patients with defective circulating CD4^+^ helper T (Th) cells have shown that CD4^+^ Th1 and Th17 cells specific to *S. aureus* are critical for host protection against infections (15, 16, 30). In this study, Th cells against *S. aureus* IsdB, a protein involved in iron acquisition from host hemoproteins and against *S. aureus* Hla were assessed. No difference in Th cells against IsdB was observed between ESRD patients and controls, whereas the percentage of individuals displaying Th cells against Hla differed between the two cohorts. At the time of inclusion, the frequency of Th cells specific to Hla tended to be lower in ESRD HD patients than in controls, indicating an impact of renal deficiency on these specific T-helper responses.

Moreover, the specific antibody and cellular responses of the three donors infected with *S. aureus* present a similar profile to that observed in other patients (data not shown). Only a few donors were infected, likely due to the progressive replacement of the central venous catheter to fistula. The low number of infected patients among the ESRD HD cohort did not allow us to draw robust conclusions. A larger-scale study including a higher number of infected patients may be more representative and highlight some differences between infected and uninfected patients. In addition, the study protocol endorsed by the ethical committee included only 3 timepoints for blood sampling (M0, M6 and M12). To optimize the analysis of the immune response induced by *S. aureus* infection in such patients, additional blood samples collected closer to the time of infection would be more relevant.

### B and T-cell decrease

All lymphocyte populations were substantially reduced, mainly B cells (60% reduction) and T cells (40% reduction) in blood from ESRD patients compared with controls.

Several studies have previously demonstrated significant B lymphopenia in ESRD patients (31–36). Here, the longitudinal and simultaneous monitoring of various immune parameters has showed that the reduction in B cell frequency was marked greater than for the other lymphocyte subsets or myeloid cells in ESRD HD patients. This reduction in B cells was maintained, or even worsened, during the one-year follow-up. In parallel, a significant decrease of total serum IgM titers, but not total IgG titers, was observed in ESRD HD cohort, suggesting a reduction of predominantly naïve B cells in this patient population. These data are in line with previous observations suggesting that naïve B cells are the most affected subpopulation in ESRD patients, due to enhanced apoptosis (32).

All T-lymphocyte subpopulations also underwent a significant and persistent decrease over one year in the ESRD HD cohort, as previously reported for naïve CD4^+^ T cells (35, 37, 38). Here, this decrease was observed for all T cell subpopulations assessed but TCRγδ T-cells were the most impacted. Although unconventional TCRγδ^+^ T cells account for 0.5% to 10% of all circulating T lymphocytes (39), they are major producers of IL-17A, TNF-α and IFNγ (40). These cytokines are involved in phagocyte activation (40, 41) and therefore contribute to the host defense against bacterial pathogens like *S. aureus* (42–44). Indeed, a concomitant and strong decrease of both CD4^+^- and TCRγδ^+^ T cells in ESRD HD patients may substantially affect host immunity (45, 46).

### B-cell number, a potential biomarker of ESRD patient outcome

According to the United States Renal Data System (USRDS) 2020 annual data report, kidney failure affects almost 750,000 people per year in the United States. Mortality after one year of HD treatment is high in ESRD patients and was estimated at 15-20% during a follow-up period from 2001 to 2016. In our small longitudinal study, the mortality rate was estimated at 13%, Among the six patients who died, three died during the first 6 months and three died between the M6 and M12 visits. As T- and B-cell numbers were markedly reduced in ESRD HD patients, the association of lymphocyte reduction with mortality was assessed, including adjustments for age and comorbidity (Charlson index). Cox proportional hazards models suggested an association between B-cell decrease and a prognosis of all-cause mortality in this ESRD HD cohort. Notably, for the three patients who died after 6 months, the drop in B-cell number progressed until death. Two recent studies have reported such an association between B-cell reduction and all-cause mortality (34, 36). Furthermore, Lin et al. have shown that a decrease in both B1- and B2-cell subpopulations was associated with mortality in CKD patients. Although the diagnosis of ESRD patient mortality has been previously associated with the decline in naïve T-cell concentrations (47–49), we failed to demonstrate an association between T-cell decline and patient mortality under our experimental conditions.

### Innate immunity

In its entirety, innate immunity was less impacted than adaptive immunity in ESRD patients although some parameters were altered. At inclusion, PMN concentration was significantly higher in ESRD HD patients than in healthy controls, albeit remaining within the normal range. These observations are in line with previous studies reporting similar increased levels in CKD patients (50, 51). After 6 months, PMN levels in the ESRD cohort were comparable to those in the controls, suggesting a beneficial effect of HD. In this study, monocyte numbers appeared unchanged in ESRD patients compared with controls, whereas recent studies have demonstrated an increase in monocyte number in CKD patients (51, 52). This discrepancy may be explained by the population size, or the early HD period selected for the ESRD patient follow-up.

In ESRD HD cohort, not only PMN concentration but also the expression of specific markers can be altered. Basal expression of the adhesion molecule CD11b was not impaired but its expression on activated phagocytes was significantly decreased in the ESRD HD cohort as previously reported (53). Moreover, CD11b expression impairment persisted throughout the one-year follow-up. Impaired expression of chemokine receptors, modulating monocyte and neutrophil functions and recruitment, could increase susceptibility to bacterial infection (54, 55). Here, the basal expression of CXCR1 on neutrophils, and CCR2 on monocytes, were impaired in ESRD HD patients. Monocyte activation resulted in a rapid downregulation of CCR2 surface expression, which was greater in ESRD HD patients than in controls. This suggests a slow down or prevention of monocyte migration to the infection site in this population (56). No difference was observed in CXCR1 expression between ESRD and controls on activated neutrophils, suggesting that PMN chemotaxis may be not impacted in ESRD HD patients.

The key role of TLR in host defense against *S. aureus* has been described in individuals with a congenital alteration in TLR signaling, in whom migration of neutrophils and monocytes to the site of infection is impaired (57). In the present study, although TLR2 and TLR4 expression was down-regulated. This down-regulation did not appear to be deleterious, since both receptors retained their ability to induce monocyte activation. Several studies have already investigated TLR2 and TLR4 expression on innate immune cells from ESRD patients, with different conclusions, probably due to the heterogenous demographic and clinical characteristics of ESRD cohorts studied (58–60).

The ability of monocytes to present antigens to CD4^+^ Th cells was assessed through the expression HLA-DR and CD86. No significant difference in CD86 expression was observed between the ESRD patients and controls. Interestingly, basal and activated expression of HLA-DR was significantly decreased in ESRD HD patients at M0, compared with controls. These results are in line with previous published data on monocytes (61) and dendritic cells (62). However, our longitudinal study showed that levels of both basal and activated HLA-DR expressions gradually returned to normal during the one-year follow-up, suggesting a beneficial effect of HD on the presentation function of monocytes.

We did not report any basal inflammation of the innate immune cells in the ESRD HD patients, contrary to published data (63). Indeed, the expression of all innate immune markers monitored in the present longitudinal study on monocytes and neutrophils was reduced at each time point and was therefore linked to a decrease rather than an increase in inflammation status. Moreover, CH50 and C3 complement molecules titrated in blood as well as inflammatory cytokines, including IL-6, TNF-α and IL-1β, in ESRD patient sera were comparable to those in healthy individual sera.

The development of vaccines against *S. aureus* infection will have to face the challenge of inducing efficient B- and T-cell responses in ESRD patients with impaired adaptive immunity. To overcome the unresponsiveness of such patients to vaccination, several protocols have already been successfully tested through increased vaccine doses and repeated vaccine injections (4). However, since innate immunity appeared to be functional in ESRD patients during the first year of HD, the use of an appropriate vaccine adjuvant could help to promote the adaptive immune response, circumventing the immunosuppression of these patients.

## Conclusion

Overall, this study showed that innate immunity appeared to be only slightly altered in ESRD HD patients during the first year of dialysis. Therefore, the early response to infection or the innate activation triggered by vaccine adjuvants is expected to be functional. On the contrary, anti-*S. aureus* adaptive immunity was significantly impaired in ESRD HD patients, in part due to markedly reduced B and T cell blood levels. Considering that B-cell compartment was the most affected, it would be of interest to investigate whether B-cell concentration could be a predictive marker of patient outcome in a larger ESRD HD population.

## Data availability statement

The raw data can be transferred to a qualified researcher by the lead/corresponding author upon request.

## Ethics statement

The studies involving human participants were reviewed and approved by the ethical committee, Comité de Protection des Personnes est-III, Hôpital de Brabois, France. The patients/participants provided their written informed consent to participate in this study.

## Author contributions

RI, BR, EB-N and SR contributed to the study concept. AD-H, SR, and EB-N contributed to experiment design. AD-H, M-JA, BB, AL and EB-N contributed to data acquisition. AD-H, NA, M-JA, BB, AL, EB-N and SR contributed to data analysis and interpretation. AD-H, NA and SR contributed to drafting this publication. AD-H, NA, BR, EB-N and SR contributed to revising this publication. All authors contributed to the article and approved the submitted version.

## References

[1] Lv JC Zhang LX. Prevalence and disease burden of chronic kidney disease. Adv Exp Med Biol (2019) 1165:3–15. doi: 10.1007/978-981-13-8871-2_1 31399958

[2] Cohen G. Immune dysfunction in uremia 2020. Toxins (2020) 12(7):439. doi: 10.3390/toxins12070439 32635646 PMC7404977

[3] Betjes MG. Uremia-associated ageing of the thymus and adaptive immune responses. Toxins (2020) 12(4):224. doi: 10.3390/toxins12040224 32260178 PMC7232426

[4] Connolly R Denton MD Humphreys H McLoughlin RM. Would hemodialysis patients benefit from a staphylococcus aureus vaccine? Kidney Int (2019) 95(3):518–25. doi: 10.1016/j.kint.2018.10.023 30691691

[5] Khan SF Bowman BT. Vaccinating the patient with eskd. Clin J Am Soc Nephrology: CJASN (2019) 14(10):1525–7. doi: 10.2215/cjn.02210219 PMC6777591 31262770

[6] Moreno NF McAdams R Goss JA Galvan NTN. Covid-19 vaccine efficacy and immunogenicity in end-stage renal disease patients and kidney transplant recipients. Curr Transplant Rep (2022) 9(3):174–84. doi: 10.1007/s40472-022-00366-1 PMC9051503 35506151

[7] Windpessl M Bruchfeld A Anders HJ Kramer H Waldman M Renia L. Covid-19 vaccines and kidney disease. Nat Rev Nephrol (2021) 17(5):291–3. doi: 10.1038/s41581-021-00406-6 PMC7869766 33558753

[8] Centers for Disease Control. Guidelines for vaccinating kidney dialysis patients and patients with chronic kidney disease. (2012). Available from: https://www.cdc.gov/vaccines/pubs/downloads/dialysis-guide-2012.pdf.

[9] Ramanathan V Winkelmayer WC. Timing of dialysis initiation–do health care setting or provider incentives matter? Clin J Am Soc Nephrol (2015) 10(8):1321–3. doi: 10.2215/cjn.07260715 PMC4527026 26206892

[10] Inrig JK Sun JL Yang Q Briley LP Szczech LA. Mortality by dialysis modality among patients who have end-stage renal disease and are awaiting renal transplantation. Clin J Am Soc Nephrology: CJASN (2006) 1(4):774–9. doi: 10.2215/cjn.00580705 17699286

[11] Nielsen LH Jensen-Fangel S Benfield T Skov R Jespersen B Larsen AR. Risk and prognosis of staphylococcus aureus bacteremia among individuals with and without end-stage renal disease: a Danish, population-based cohort study. BMC Infect Dis (2015) 15:6. doi: 10.1186/s12879-014-0740-8 25566857 PMC4296555

[12] Vanegas JM Salazar-Ospina L Gallego MA Jiménez JN. A longitudinal study shows intermittent colonization by staphylococcus aureus with a high genetic diversity in hemodialysis patients. Int J Med microbiology: IJMM (2021) 311(1):151471. doi: 10.1016/j.ijmm.2020.151471 33373839

[13] Minegishi Y Saito M Nagasawa M Takada H Hara T Tsuchiya S. Molecular explanation for the contradiction between systemic Th17 defect and localized bacterial infection in hyper-ige syndrome. J Exp Med (2009) 206(6):1291–301. doi: 10.1084/jem.20082767 PMC2715068 19487419

[14] Ishigame H Kakuta S Nagai T Kadoki M Nambu A Komiyama Y. Differential roles of interleukin-17a and -17f in host defense against mucoepithelial bacterial infection and allergic responses. Immunity (2009) 30(1):108–19. doi: 10.1016/j.immuni.2008.11.009 19144317

[15] Brown AF Murphy AG Lalor SJ Leech JM O’Keeffe KM Mac Aogáin M. Memory Th1 cells are protective in invasive staphylococcus aureus infection. PloS Pathog (2015) 11(11):e1005226. doi: 10.1371/journal.ppat.1005226 26539822 PMC4634925

[16] Zielinski CE Mele F Aschenbrenner D Jarrossay D Ronchi F Gattorno M. Pathogen-induced human Th17 cells produce ifn-Γ or il-10 and are regulated by il-1β. Nature (2012) 484(7395):514–8. doi: 10.1038/nature10957 22466287

[17] Kim MH Granick JL Kwok C Walker NJ Borjesson DL Curry FR. Neutrophil survival and c-Kit(+)-Progenitor proliferation in staphylococcus aureus-infected skin wounds promote resolution. Blood (2011) 117(12):3343–52. doi: 10.1182/blood-2010-07-296970 PMC3069674 21278352

[18] van den Berg JM van Koppen E Ahlin A Belohradsky BH Bernatowska E Corbeel L. Chronic granulomatous disease: the European experience. PloS One (2009) 4(4):e5234. doi: 10.1371/journal.pone.0005234 19381301 PMC2668749

[19] Lê Cao K-A Boitard S Besse P. Sparse pls discriminant analysis: Biologically relevant feature selection and graphical displays for multiclass problems. BMC Bioinf (2011) 12(1):253. doi: 10.1186/1471-2105-12-253 PMC3133555 21693065

[20] Nguyen DV Rocke DM. Tumor classification by partial least squares using microarray gene expression data. Bioinformatics (2002) 18(1):39–50. doi: 10.1093/bioinformatics/18.1.39 11836210

[21] Pérez-Enciso M Tenenhaus M. Prediction of clinical outcome with microarray data: a partial least squares discriminant analysis (Pls-da) approach. Hum Genet (2003) 112(5-6):581–92. doi: 10.1007/s00439-003-0921-9 12607117

[22] van Kessel KP Bestebroer J van Strijp JA. Neutrophil-mediated phagocytosis of staphylococcus aureus. Front Immunol (2014) 5:467. doi: 10.3389/fimmu.2014.00467 25309547 PMC4176147

[23] Andrews T Sullivan KE. Infections in patients with inherited defects in phagocytic function. Clin Microbiol Rev (2003) 16(4):597–621. doi: 10.1128/cmr.16.4.597-621.2003 14557288 PMC207096

[24] Miller LS Cho JS. Immunity against staphylococcus aureus cutaneous infections. Nat Rev Immunol (2011) 11(8):505–18. doi: 10.1038/nri3010 PMC5868361 21720387

[25] Haag-Weber M Hörl WH. Dysfunction of polymorphonuclear leukocytes in uremia. Semin Nephrol (1996) 16(3):192–201. 8734462

[26] Sardenberg C Suassuna P Andreoli MC Watanabe R Dalboni MA Manfredi SR. Effects of uraemia and dialysis modality on polymorphonuclear cell apoptosis and function. Nephrology dialysis transplantation: Off Publ Eur Dialysis Transplant Assoc - Eur Renal Assoc (2006) 21(1):160–5. doi: 10.1093/ndt/gfi095 16155068

[27] Ragle BE Bubeck Wardenburg J. Anti-Alpha-Hemolysin monoclonal antibodies mediate protection against staphylococcus aureus pneumonia. Infection Immun (2009) 77(7):2712–8. doi: 10.1128/iai.00115-09 PMC2708543 19380475

[28] Pilsczek FH Salina D Poon KK Fahey C Yipp BG Sibley CD. A novel mechanism of rapid nuclear neutrophil extracellular trap formation in response to staphylococcus aureus. J Immunol (2010) 185(12):7413–25. doi: 10.4049/jimmunol.1000675 21098229

[29] Kim JK Lee HW Joo N Lee HS Song YR Kim HJ. Prognostic role of circulating neutrophil extracellular traps levels for long-term mortality in new end-stage renal disease patients. Clin Immunol (2020) 210:108263. doi: 10.1016/j.clim.2019.108263 31629808

[30] Utay NS Roque A Timmer JK Morcock DR DeLeage C Somasunderam A. Mrsa infections in hiv-infected people are associated with decreased mrsa-specific Th1 immunity. PloS Pathog (2016) 12(4):e1005580. doi: 10.1371/journal.ppat.1005580 27093273 PMC4836670

[31] Fernández-Fresnedo G Ramos MA González-Pardo MC de Francisco AL López-Hoyos M Arias M. B lymphopenia in uremia is related to an accelerated in vitro apoptosis and dysregulation of bcl-2. Nephrology dialysis transplantation: Off Publ Eur Dialysis Transplant Assoc - Eur Renal Assoc (2000) 15(4):502–10. doi: 10.1093/ndt/15.4.502 10727545

[32] Pahl MV Gollapudi S Sepassi L Gollapudi P Elahimehr R Vaziri ND. Effect of end-stage renal disease on b-lymphocyte subpopulations, il-7, baff and baff receptor expression. Nephrology dialysis transplantation: Off Publ Eur Dialysis Transplant Assoc - Eur Renal Assoc (2010) 25(1):205–12. doi: 10.1093/ndt/gfp397 PMC2796898 19684120

[33] Saad K Elsayh KI Zahran AM Sobhy KM. Lymphocyte populations and apoptosis of peripheral blood b and T lymphocytes in children with end stage renal disease. Renal failure (2014) 36(4):502–7. doi: 10.3109/0886022x.2013.875833 24512046

[34] Molina M Allende LM Ramos LE Gutiérrez E Pleguezuelo DE Hernández ER. Cd19(+) b-cells, a new biomarker of mortality in hemodialysis patients. Front Immunol (2018) 9:1221. doi: 10.3389/fimmu.2018.01221 29963040 PMC6013647

[35] Freitas GRR da Luz Fernandes M Agena F Jaluul O Silva SC Lemos FBC. Aging and end stage renal disease cause a decrease in absolute circulating lymphocyte counts with a shift to a memory profile and diverge in treg population. Aging Dis (2019) 10(1):49–61. doi: 10.14336/ad.2018.0318 30705767 PMC6345336

[36] Lin J Tang W Liu W Yu F Wu Y Fang X. Decreased B1 and B2 lymphocytes are associated with mortality in elderly patients with chronic kidney diseases. Front Med (2020) 7:75. doi: 10.3389/fmed.2020.00075 PMC7098909 32266271

[37] Chiu YL Shu KH Yang FJ Chou TY Chen PM Lay FY. A comprehensive characterization of aggravated aging-related changes in T lymphocytes and monocytes in end-stage renal disease: the iesrd study. Immun ageing: I A (2018) 15:27. doi: 10.1186/s12979-018-0131-x 30455721 PMC6223078

[38] Xiang FF Zhu JM Cao XS Shen B Zou JZ Liu ZH. Lymphocyte depletion and subset alteration correlate to renal function in chronic kidney disease patients. Renal failure (2016) 38(1):7–14. doi: 10.3109/0886022x.2015.1106871 26539739

[39] Garcillán B Marin AV Jiménez-Reinoso A Briones AC Muñoz-Ruiz M García-León MJ. Γδ T lymphocytes in the diagnosis of human T cell receptor immunodeficiencies. Front Immunol (2015) 6:20. doi: 10.3389/fimmu.2015.00020 25688246 PMC4310324

[40] Griffin GK Newton G Tarrio ML Bu DX Maganto-Garcia E Azcutia V. Il-17 and tnf-α sustain neutrophil recruitment during inflammation through synergistic effects on endothelial activation. J Immunol (2012) 188(12):6287–99. doi: 10.4049/jimmunol.1200385 PMC3370121 22566565

[41] Zhang Y Wang H Ren J Tang X Jing Y Xing D. Il-17a synergizes with ifn-Γ to upregulate inos and no production and inhibit chlamydial growth. PloS One (2012) 7(6):e39214. doi: 10.1371/journal.pone.0039214 22745717 PMC3379979

[42] Marchitto MC Dillen CA Liu H Miller RJ Archer NK Ortines RV. Clonal Vγ6(+)Vδ4(+) T cells promote il-17-Mediated immunity against staphylococcus aureus skin infection. Proc Natl Acad Sci U.S.A. (2019) 116(22):10917–26. doi: 10.1073/pnas.1818256116 PMC6561199 31088972

[43] Dillen CA Pinsker BL Marusina AI Merleev AA Farber ON Liu H. Clonally expanded Γδ T cells protect against staphylococcus aureus skin reinfection. J Clin Invest (2018) 128(3):1026–42. doi: 10.1172/jci96481 PMC5824877 29400698

[44] Hendriks A Mnich ME Clemente B Cruz AR Tavarini S Bagnoli F. Staphylococcus aureus-specific tissue-resident memory Cd4+ T cells are abundant in healthy human skin. Front Immunol (2021) 12:642711. doi: 10.3389/fimmu.2021.642711 33796109 PMC8008074

[45] Pinheiro MB Antonelli LR Sathler-Avelar R Vitelli-Avelar DM Spindola-de-Miranda S Guimarães TMPD. Cd4-Cd8-αβ and Γδ T cells display inflammatory and regulatory potentials during human tuberculosis. PloS One (2012) 7(12):e50923. doi: 10.1371/journal.pone.0050923 23239994 PMC3519797

[46] Cheng P Liu T Zhou W-Y Zhuang Y Peng L-s Zhang J-y. Role of gamma-delta T cells in host response against staphylococcus aureus-induced pneumonia. BMC Immunol (2012) 13(1):38. doi: 10.1186/1471-2172-13-38 22776294 PMC3524664

[47] Courivaud C Bamoulid J Crepin T Gaiffe E Laheurte C Saas P. Pre-transplant thymic function predicts is associated with patient death after kidney transplantation. Front Immunol (2020) 11:1653. doi: 10.3389/fimmu.2020.01653 32903778 PMC7438875

[48] Crépin T Legendre M Carron C Vachey C Courivaud C Rebibou JM. Uraemia-induced immune senescence and clinical outcomes in chronic kidney disease patients. Nephrology dialysis transplantation: Off Publ Eur Dialysis Transplant Assoc - Eur Renal Assoc (2020) 35(4):624–32. doi: 10.1093/ndt/gfy276 30202981

[49] Xiang F Chen R Cao X Shen B Chen X Ding X. Premature aging of circulating T cells predicts all-cause mortality in hemodialysis patients. BMC Nephrol (2020) 21(1):271. doi: 10.1186/s12882-020-01920-8 32660510 PMC7359274

[50] Sela S Shurtz-Swirski R Cohen-Mazor M Mazor R Chezar J Shapiro G. Primed peripheral polymorphonuclear leukocyte: a culprit underlying chronic low-grade inflammation and systemic oxidative stress in chronic kidney disease. J Am Soc Nephrology: JASN (2005) 16(8):2431–8. doi: 10.1681/asn.2004110929 15987755

[51] Naicker SD Cormican S Griffin TP Maretto S Martin WP Ferguson JP. Chronic kidney disease severity is associated with selective expansion of a distinctive intermediate monocyte subpopulation. Front Immunol (2018) 9:2845. doi: 10.3389/fimmu.2018.02845 30619252 PMC6302774

[52] Bowe B Xie Y Xian H Li T Al-Aly Z. Association between monocyte count and risk of incident ckd and progression to esrd. Clin J Am Soc Nephrology: CJASN (2017) 12(4):603–13. doi: 10.2215/cjn.09710916 PMC5383390 28348030

[53] Dadfar E Lundahl J Jacobson SH. Granulocyte extravasation and recruitment to sites of interstitial inflammation in patients with renal failure. Am J Nephrol (2004) 24(3):330–9. doi: 10.1159/000078743 15166474

[54] Maus U von Grote K Kuziel WA Mack M Miller EJ Cihak J. The role of cc chemokine receptor 2 in alveolar monocyte and neutrophil immigration in intact mice. Am J Respir Crit Care Med (2002) 166(3):268–73. doi: 10.1164/rccm.2112012 12153956

[55] Moser B Wolf M Walz A Loetscher P. Chemokines: multiple levels of leukocyte migration control. Trends Immunol (2004) 25(2):75–84. doi: 10.1016/j.it.2003.12.005 15102366

[56] Parker LC Whyte MK Vogel SN Dower SK Sabroe I. Toll-like receptor (Tlr)2 and Tlr4 agonists regulate ccr expression in human monocytic cells. J Immunol (2004) 172(8):4977–86. doi: 10.4049/jimmunol.172.8.4977 15067079

[57] Bouma G Ancliff PJ Thrasher AJ Burns SO. Recent advances in the understanding of genetic defects of neutrophil number and function. Br J haematology (2010) 151(4):312–26. doi: 10.1111/j.1365-2141.2010.08361.x 20813010

[58] Kuroki Y Tsuchida K Go I Aoyama M Naganuma T Takemoto Y. A study of innate immunity in patients with end-stage renal disease: special reference to toll-like receptor-2 and -4 expression in peripheral blood monocytes of hemodialysis patients. Int J Mol Med (2007) 19(5):783–90. doi: 10.3892/ijmm.19.5.783 17390084

[59] Koc M Toprak A Arikan H Odabasi Z Elbir Y Tulunay A. Toll-like receptor expression in monocytes in patients with chronic kidney disease and haemodialysis: Relation with inflammation. Nephrology dialysis transplantation: Off Publ Eur Dialysis Transplant Assoc - Eur Renal Assoc (2011) 26(3):955–63. doi: 10.1093/ndt/gfq500 20729266

[60] Gollapudi P Yoon JW Gollapudi S Pahl MV Vaziri ND. Leukocyte toll-like receptor expression in end-stage kidney disease. Am J Nephrol (2010) 31(3):247–54. doi: 10.1159/000276764 20090311

[61] de Cal M Cruz DN Corradi V Nalesso F Polanco N Lentini P. Hla-Dr expression and apoptosis: a cross-sectional controlled study in hemodialysis and peritoneal dialysis patients. Blood purification (2008) 26(3):249–54. doi: 10.1159/000122110 18376106

[62] Agrawal S Gollapudi P Elahimehr R Pahl MV Vaziri ND. Effects of end-stage renal disease and haemodialysis on dendritic cell subsets and basal and lps-stimulated cytokine production. Nephrology dialysis transplantation: Off Publ Eur Dialysis Transplant Assoc - Eur Renal Assoc (2009) 25(3):737–46. doi: 10.1093/ndt/gfp580 19903659

[63] Tang PC Zhang YY Chan MK Lam WW Chung JY Kang W. The emerging role of innate immunity in chronic kidney diseases. Int J Mol Sci (2020) 21(11):4018. doi: 10.3390/ijms21114018 32512831 PMC7312694

