# Supplementary material for: Anti-staphylococcus aureus adaptive immunity is impaired in end-stage renal disease patients on hemodialysis: one-year longitudinal study
Source: Front Immunol. 2023 May 25;14:1123160. doi: 10.3389/fimmu.2023.1123160 (PMC10250961; doi:10.3389/fimmu.2023.1123160)
Supplement: Supplementary file 1 [file Table_1.docx]

**Supplementary material**

**Table S1. Correlation between age and immunological parameters**

***Spearman correlation coefficients (calculated at M0, M6 and M12)***

| **Immunological parameter** | **M0** | **M6** | **M12** |  |  |
| --- | --- | --- | --- | --- | --- |
| IgG_anti-IsdB | -0,279 | -0,399 | -0,382 |  | 1 |
| Oxidative burst with autologous sera | -0,391 | -0,367 | -0,432 |  | 0,5 |
| Oxidative burst with inactivated autologous sera | -0,257 | -0,357 | -0,360 |  | 0 |
| Oxidative burst with healthy sera | 0,027 | -0,059 | 0,167 |  | -0,5 |
| Oxidative burst with inactivated healthy sera | 0,102 | -0,040 | -0,157 |  | -1 |
| Bacteria survival in blood | -0,136 | -0,156 | -0,070 |  |  |
| Total IgA | -0,191 | -0,124 | -0,068 |  |  |
| Total IgG | -0,329 | -0,395 | -0,223 |  |  |
| Total IgM | -0,302 | -0,266 | -0,166 |  |  |
| Leucocytes | 0,068 | -0,095 | -0,048 |  |  |
| Monocytes | 0,036 | 0,066 | -0,134 |  |  |
| Neutrophils | 0,154 | -0,019 | 0,021 |  |  |
| Lymphocytes | -0,296 | -0,300 | -0,213 |  |  |
| B-Lymphocytes | -0,264 | -0,256 | -0,074 |  |  |
| T-Lymphocytes | -0,398 | -0,399 | -0,272 |  |  |
| NK lymphocytes | 0,192 | 0,133 | 0,058 |  |  |
| NKT lymphocytes | -0,110 | -0,134 | -0,166 |  |  |
| CD4^+^ T-cells | -0,331 | -0,302 | -0,103 |  |  |
| TCRγδ T-cells | -0,103 | -0,039 | -0,249 |  |  |
| CD8^+^ T-cells | -0,373 | -0,410 | -0,336 |  |  |
| Basal CD86 | 0,092 | -0,079 | -0,205 |  |  |
| Activated CD86 | 0,122 | 0,241 | 0,152 |  |  |
| Basal CCR2 | -0,075 | 0,220 | 0,139 |  |  |
| Activated CCR2 | 0,146 | 0,273 | 0,294 |  |  |
| Basal TLR2 | 0,140 | 0,005 | 0,065 |  |  |
| Activated TLR2 | 0,114 | -0,083 | 0,099 |  |  |
| Basal TLR4 | -0,183 | -0,069 | -0,053 |  |  |
| Activated TLR4 | -0,160 | -0,020 | 0,188 |  |  |
| Basal HLADR | 0,288 | -0,367 | -0,238 |  |  |
| Activated HLADR | 0,153 | -0,145 | -0,046 |  |  |
| Basal CD11b | 0,138 | -0,059 | 0,242 |  |  |
| Activated CD11b | -0,020 | -0,119 | 0,151 |  |  |
| Basal CD35 | 0,023 | 0,086 | 0,044 |  |  |
| Activated CD35 | 0,023 | 0,218 | 0,148 |  |  |
| Basal CXCR1 | 0,155 | 0,136 | 0,024 |  |  |
| Activated CXCR1 | 0,278 | 0,113 | 0,210 |  |  |
| Basal CD16 | 0,158 | -0,158 | 0,008 |  |  |
| Activated CD16 | 0,126 | 0,060 | 0,286 |  |  |
| IL1β | -0,095 | 0,064 | 0,039 |  |  |
| IL10 | -0,335 | -0,095 | -0,189 |  |  |
| TNFα | -0,332 | -0,071 | -0,108 |  |  |

**Table S2. Univariate analyses: comparison of control group versus ESRD groups**

S: statistically significant, NS: Non-statistically significant, > indicates group A has higher levels than group B, 1.5x indicates by how many folds are levels of group A higher than group B. < indicates group A has lower levels than group B, /1.2 indicates by how many folds are levels of group A lower than group A.

| **Variable** | **Controls vs ESRD on M0** | **Controls vs ESRD on M6** | **Controls vs ESRD on M12** |
| --- | --- | --- | --- |
| T-response anti-Hla | 0.141 (NS) | 0.570 (NS) | 0.648 (NS) |
| T-response anti-IsdB | 0.615 (NS) | 1 (NS) | 0.741 (NS) |
| IgG_anti-IsdB | **0.003 (S,>) x2.3** | 0.058 (NS) x2 | 0.258 (NS) x1.5 |
| IgG_anti-Hla | **0.007 (S,>) x2.5** | **0.032 (S,>) x2.3** | 0.082 (NS) x2.1 |
| Oxidative burst with autologous sera | **<0.001 (S)** | **<0.001 (S)** | **<0.001 (S)** |
| Oxidative burst with inactivated autologous sera | **0.001 (S)** | **0.001 (S)** | **0.036 (S)** |
| Oxidative burst with healthy sera | 0.257 (NS) | 0.297 (NS) | 0.297 (NS) |
| Oxidative burst with inactivated healthy sera | 0.941 (NS) x1 | 1 (NS) x1 | 1 (NS) x1 |
| Bacteria survival in blood | **0.049 (S)** | 0.261 (NS) | 0.113 (NS) |
| Total IgA | 0.765 (NS) /1.1 | 0.641 (NS) /1.2 | 0.697 (NS) /1.2 |
| Total IgG | 0.117(NS) | 0.444 (NS) | 0.277 (NS) |
| Total IgM | **0.004 (S,>) x1.9** | **0.009 (S,>) x1.9** | **0.036 (S,>) x1.7** |
| Leucocytes | 0.475 (NS) /1.1 | 1 (NS) x1 | 0.925 (NS) /1.1 |
| Monocytes | 0.765 (NS) /1.1 | 1 (NS) /1 | 1 (NS) x1 |
| Neutrophils | **0.006 (S,<) /1.4** | 0.364 (NS) /1.2 | 0.064 (NS) /1.3 |
| Lymphocytes | **<0.001 (S,>) x1.6** | **<0.001 (S,>) x1.6** | **0.002 (S,>) x1.5** |
| B-Lymphocytes | **<0.001 (S,>) x3.1** | **<0.001 (S,>) x3** | **0.001 (S,>) x2.6** |
| T-Lymphocytes | **<0.001 (S,>) x1.6** | **<0.001 (S,>) x1.7** | **<0.001 (S,>) x1.7** |
| NK lymphocytes | **0.013 (S,>) x1.5** | 0.311 (NS) x1.3 | 1 (NS) x1.1 |
| NKT lymphocytes | 0.140 (NS) x1.5 | 0.131 (NS) x1.5 | 0.125 (NS) x1.6 |
| CD4^+^ T-cells | **<0.001 (S,>) x1.6** | **<0.001 (S,>) x1.6** | **0.005 (S,>) x1.6** |
| TCRγδ T-cells | **<0.001 (S,>) x2.4** | **<0.001 (S,>) x2.5** | **<0.001 (S,>) x3.1** |
| CD8^+^ T-cells | **0.003 (S,>) x1.6** | **0.001 (S,>) x1.7** | **<0.001 (S,>) x1.8** |
| Basal CD86 | 0.684 (NS) x1.1 | 1 (NS) /1 | 1 (NS) x1 |
| Activated CD86 | 0.475 (NS) /1.1 | 0.092 (NS) /1.2 | 0.491 (NS) /1.1 |
| Basal CCR2 | 0.613 (NS) x1.2 | 1 (NS) /1.1 | 1 (NS) /1 |
| Activated CCR2 | **<0.001 (S)** | **0.001 (S)** | 0.064 (NS) |
| Basal TLR2 | **0.035 (S,>) x1.2** | **0.032 (S,>) x1.2** | 0.380 (NS) x1.1 |
| Activated TLR2 | **0.018 (S,>) x1.2** | **0.002 (S,>) x1.3** | **0.036 (S,>) x1.2** |
| Basal TLR4 | 0.3 (NS) | 0.344 (NS) | 0.098 (NS) |
| Activated TLR4 | 0.419 (NS) | 0.444 (NS) | 0.064 (NS) |
| Basal HLADR | **0.001 (S,>) x1.6** | 0.164 (NS) x1.3 | 1 (NS) /1 |
| Activated HLADR | **<0.001 (S,>) x2** | **0.018 (S,>) x1.4** | 0.925 (NS) x1.1 |
| Basal CD11b | 0.995 (NS) | 1 (NS) | 1 (NS) |
| Activated CD11b | **0.024 (S,>) x1.3** | 0.059 (NS) x1.3 | **0.002 (S,>) x1.5** |
| Basal CD35 | 0.220 (NS) /1.1 | 1 (NS) /1 | 0.943 (NS) /1.1 |
| Activated CD35 | 0.755 (NS) /1.1 | 1 (NS) /1 | 0.821 (NS) x1.1 |
| Basal CXCR1 | 0.258 (NS) x1.1 | 1 (NS) /1 | 1 (NS) /1 |
| Activated CXCR1 | 0.995 (NS) /1 | 1 (NS) /1.1 | 1 (NS) /1.1 |
| Basal CD16 | **0.021 (S,>) x1.2** | 0.073 (NS) x1.2 | 0.130 (NS) x1.2 |
| Activated CD16 | 0.306 (NS) x1.1 | 0.286 (NS) x1.1 | 0.239 (NS) x1.1 |
| CCR2 ratio* | 0.572 (NS) /1.2 | 0.203 (NS) /1.3 | 0.830 (NS) /1.1 |
| TLR2 ratio* | 0.995 (NS) /1 | 1 (NS) x1 | 0.925 (NS) x1.1 |
| HLA_DR ratio* | **0.045 (S,>) x1.3** | 0.711 (NS) x1.1 | 0.775 (NS) x1.1 |
| CD11b ratio* | **0.015 (S,>) x1.2** | 0.057 (NS) x1.2 | 0.124 (NS) x1.9 |
| CD35 ratio* | 0.762 (NS) | 0.931 (NS) | 0.098 (NS) |
| CD16 ratio* | 0.536 (NS) /1.1 | 1 (NS) /1 | 0.697 (NS) x1.4 |
| IL1β | 0.175 (NS) x1.8 | 1 (NS) x1.2 | 0.943 (NS) x1.2 |
| IL10 | 0.980 (NS) x1.1 | 1 (NS) x1 | 1 (NS) x1.1 |
| TNFα | 0.995 (NS) x1.1 | 0.444 (NS) /1.4 | 1 (NS) /1.2 |

**Ratio:* MFI (basal marker) /MFI (activated marker)

**Table S3. Univariate analyses: comparison of native versus inactivated sera in oxidative burst:**

The levels of oxidative burst were compared between native and heat-inactivated sera for each group separately (controls, ESRD M0, ESRD M6, ESRD M12). Paired t-tests were used for normally distributed variables and a Wilcoxon signed rank test for non-normally distributed variables. No correction for multiple testing was performed as the number of tests for this sub-analysis was low.

S: statistically significant, NS: Non-statistically significant, > indicates group A has higher levels than group B, 1.5x indicates by how many folds are levels of group B higher than group A.

| **Variable** | **AS vs IAS** |
| --- | --- |
| Controls | **0.001 (S)** |
| ESRD M0 | 0.768 (NS) /2 |
| ESRD M12 | 0.410 (NS) x6.8 |
| ESRD M6 | 0.059 (NS) x87.2 |

*AS: Autologous serum*

*IAS: Inactivated autologous serum*

**Table S4. Univariate analyses: comparison of ESRD groups**

S: statistically significant, NS: Non-statistically significant, > indicates group A has higher levels than group B, 1.5x indicates by how many folds are levels of group A higher than group B. < indicates group A has lower levels than group B, /1.2 indicates by how many folds are levels of group A lower than group A.

| **Variable** | **ESRD on M0 vs ESRD on M6** | **ESRD on M0 vs ESRD on M12** | **ESRD on M6 vs ESRD on M12** |
| --- | --- | --- | --- |
| T-response anti-Hla | 0.997 (NS) | 0.915 (NS) | 1 (NS) |
| T-response anti-IsdB | 0.992 (NS) | 0.986 (NS) | 1 (NS) |
| IgG_anti-IsdB | 0.992 (NS) /1.1 | 0.986 (NS) /1.5 | 1 (NS) /1.3 |
| IgG_anti-Hla | 0.992 (NS) /1.1 | 0.986 (NS) /1.2 | 1 (NS) /1.1 |
| Oxidative burst with autologous sera | 0.992 (NS) | 0.986 (NS) | 1 (NS) |
| Oxidative burst with inactivated autologous sera | 0.992 (NS) | 0.986 (NS) | 1 (NS) |
| Oxidative burst with healthy sera | 0.992 (NS) | 0.986 (NS) | 1 (NS) |
| Oxidative burst with inactivated healthy sera | 0.991 (NS) /1 | 0.985 (NS) /1 | 1 (NS) x1 |
| Bacteria survival in blood | 0.991 (NS) | 0.985 (NS) | 1 (NS) |
| Total IgA | 0.991 (NS) /1.1 | 0.985 (NS) /1.1 | 1 (NS) x1 |
| Total IgG | 0.991 (NS) | 0.985 (NS) | 1 (NS) |
| Total IgM | 0.991 (NS) /1 | 0.985 (NS) /1.1 | 1 (NS) /1.1 |
| Leucocytes | 0.991 (NS) x1.1 | 0.985 (NS) x1 | 1 (NS) /1.1 |
| Monocytes | 0.991 (NS) x1.1 | 0.985 (NS) x1.1 | 1 (NS) x1 |
| Neutrophils | 0.991 (NS) x1.2 | 0.985 (NS) x1.1 | 1 (NS) /1.1 |
| Lymphocytes | 0.991 (NS) x1 | 0.985 (NS) /1 | 1 (NS) /1.1 |
| B-Lymphocytes | 0.991 (NS) /1.1 | 0.985 (NS) /1.2 | 1 (NS) /1.2 |
| T-Lymphocytes | 0.991 (NS) x1 | 0.985 (NS) x1 | 1 (NS) /1 |
| NK lymphocytes | 0.991 (NS) /1.2 | 0.713 (NS) /1.4 | 1 (NS) /1.2 |
| NKT lymphocytes | 0.991 (NS) x1 | 0.985 (NS) x1.1 | 1 (NS) x1 |
| CD4^+^ T-cells | 0.991 (NS) x1 | 0.985 (NS) /1 | 1 (NS) /1.1 |
| TCRγδ T-cells | 0.991 (NS) x1 | 0.985 (NS) x1.3 | 1 (NS) x1.2 |
| CD8^+^ T-cells | 0.991 (NS) x1.1 | 0.985 (NS) x1.1 | 1 (NS) x1.1 |
| Basal CD86 | 0.991 (NS) /1.1 | 0.985 (NS) /1 | 1 (NS) x1.1 |
| Activated CD86 | 0.991 (NS) /1.1 | 0.985 (NS) /1 | 1 (NS) x1.1 |
| Basal CCR2 | 0.991 (NS) /1.2 | 0.985 (NS) /1.2 | 1 (NS) x1 |
| Activated CCR2 | **0.047 (S)** | **<0.001 (S)** | 1 (NS) |
| Basal TLR2 | 0.991 (NS) x1 | 0.985 (NS) /1.1 | 1 (NS) /1.1 |
| Activated TLR2 | 0.991 (NS) x1.1 | 0.985 (NS) x1 | 1 (NS) /1 |
| Basal TLR4 | 0.244 (NS) | **0.001 (S)** | 1 (NS) |
| Activated TLR4 | 0.244 (NS) | **0.001 (S)** | 1 (NS) |
| Basal HLADR | 0.991 (NS) /1.2 | **0.007 (S,<) /1.6** | 1 (NS) /1.3 |
| Activated HLADR | 0.244 (NS) /1.4 | **<0.001 (S,<) /1.8** | 1 (NS) /1.3 |
| Basal CD11b | 0.991 (NS) | 0.985 (NS) | 1 (NS) |
| Activated CD11b | 0.991 (NS) /1 | 0.985 (NS) x1.1 | 1 (NS) x1.1 |
| Basal CD35 | 0.991 (NS) x1.1 | 0.985 (NS) x1.1 | 1 (NS) /1 |
| Activated CD35 | 0.991 (NS) x1 | 0.861 (NS) x1.2 | 1 (NS) x1.1 |
| Basal CXCR1 | 0.991 (NS) /1.1 | 0.915 (NS) /1.1 | 1 (NS) /1 |
| Activated CXCR1 | 0.991 (NS) /1.1 | 0.985 (NS) /1.1 | 1 (NS) x1 |
| Basal CD16 | 0.991 (NS) /1 | 0.985 (NS) /1 | 1 (NS) /1 |
| Activated CD16 | 0.991 (NS) x1 | 0.985 (NS) x1 | 1 (NS) x1 |
| CCR2 ratio* | 0.991 (NS) /1.1 | 0.985 (NS) x1 | 1 (NS) x1.1 |
| TLR2 ratio* | 0.991 (NS) x1 | 0.985 (NS) x1.1 | 1 (NS) x1 |
| HLA_DR ratio* | 0.991 (NS) /1.2 | 0.985 (NS) /1.2 | 1 (NS) x1 |
| CD11b ratio* | 0.991 (NS) /1 | 0.915 (NS) x1.6 | 1 (NS) x1.6 |
| CD35 ratio* | 0.991 (NS) | 0.861 (NS) | 1 (NS) |
| CD16 ratio* | 0.991 (NS) x1 | 0.915 (NS) x1.5 | 1 (NS) x1.5 |
| IL1β | 0.991 (NS) /1.5 | 0.915 (NS) /1.5 | 1 (NS) /1 |
| IL10 | 0.991 (NS) /1.1 | 0.985 (NS) /1 | 1 (NS) x1.1 |
| TNFα | 0.804 (NS) /1.6 | 0.985 (NS) /1.3 | 1 (NS) x1.2 |

**Ratio:* MFI (basal marker) /MFI (activated marker)

**Table S5. Univariate analyses: longitudinal comparison of ESRD groups**

*Group comparisons computed on the ESRD subset from longitudinal model*

This analysis was limited to ESRD subjects with available values at all 3 time points (M0, M6, M12). Each biological parameter was examined in one-factor ANOVA analysis (with a repeated statement where needed to account for group heterogeneity) with age included in the model as an adjustment covariate or a Wilcoxon sum rank test performed on age residuals of each variable, depending on variables’ distribution. Pairwise time point comparisons were computed from these models. to account for multiple testing a Tukey adjustment was used in parametric analyses and an FDR correction in the non-parametric analyses. Given that more than 50 variables were assessed, to further control for false positive results an FDR correction was applied across all variables for each type of comparison.

S: statistically significant, NS: Non-statistically significant, > indicates group A has higher levels than group B, 1.5x indicates by how many folds are levels of group A higher than group B. < indicates group A has lower levels than group B, /1.2 indicates by how many folds are levels of group A lower than group A.

| **Variable** | **ESRD on M0 vs ESRD on M6** | **ESRD on M0 vs ESRD on M12** | **ESRD on M6 vs ESRD on M12** |
| --- | --- | --- | --- |
| T-response anti-Hla | 1 (NS) | 0.872 (NS) | 1 (NS) |
| T-response anti-IsdB | 1 (NS) /1.4 | 0.998 (NS) x1.1 | 1 (NS) x1.6 |
| IgG_anti-IsdB | 1 (NS) /1.1 | 0.998 (NS) /1.3 | 1 (NS) /1.2 |
| IgG_anti-Hla | 1 (NS) x1 | 0.998 (NS) x1 | 1 (NS) x1 |
| Oxidative burst with autologous sera | 1 (NS) /1 | 0.998 (NS) /1.1 | 1 (NS) /1.1 |
| Oxidative burst with inactivated autologous sera | 1 (NS) | 0.998 (NS) | 1 (NS) |
| Oxidative burst with healthy sea | 1 (NS) x1 | 0.998 (NS) /1 | 1 (NS) /1 |
| Oxidative burst with inactivated healthy sera | 1 (NS) | 0.998 (NS) | 1 (NS) |
| Bacteria survival in blood | 1 (NS) | 0.998 (NS) | 1 (NS) |
| Total IgA | 1 (NS) /1 | 0.998 (NS) /1 | 1 (NS) /1 |
| Total IgG | 1 (NS) | 0.998 (NS) | 1 (NS) |
| Total IgM | 1 (NS) /1 | 0.998 (NS) /1.1 | 1 (NS) /1.1 |
| Leucocytes | 1 (NS) | 0.998 (NS) | 1 (NS) |
| Monocytes | 1 (NS) x1.1 | 0.998 (NS) x1.1 | 1 (NS) x1 |
| Neutrophils | 1 (NS) x1.2 | 0.998 (NS) x1.1 | 1 (NS) /1.1 |
| Lymphocytes | 1 (NS) x1.1 | 0.998 (NS) /1 | 1 (NS) /1.1 |
| B-Lymphocytes | 1 (NS) x1.1 | 0.998 (NS) /1 | 1 (NS) /1.1 |
| T-Lymphocytes | 1 (NS) x1.1 | 0.998 (NS) x1 | 1 (NS) /1.1 |
| NK lymphocytes | 1 (NS) /1.1 | 0.872 (NS) /1.3 | 1 (NS) /1.2 |
| NKT lymphocytes | 1 (NS) x1.1 | 0.998 (NS) x1 | 1 (NS) /1.1 |
| CD4^+^ T-cells | 1 (NS) x1 | 0.998 (NS) /1 | 1 (NS) /1.1 |
| TCRγδ T-cells | 1 (NS) x1 | 0.998 (NS) x1.2 | 1 (NS) x1.2 |
| CD8^+^ T-cells | 1 (NS) x1.2 | 0.998 (NS) x1.1 | 1 (NS) /1 |
| Basal CD86 | 1 (NS) /1 | 0.998 (NS) x1.1 | 1 (NS) x1.1 |
| Activated CD86 | 1 (NS) x1 | 0.998 (NS) x1.1 | 1 (NS) x1 |
| Basal CCR2 | 1 (NS) /1.3 | 0.998 (NS) /1.1 | 1 (NS) x1.1 |
| Activated CCR2 | 0.754 (NS) /1.1 | **0.006 (S,<) /1.2** | 1 (NS) /1.1 |
| Basal TLR2 | 1 (NS) x1 | 0.998 (NS) /1 | 1 (NS) /1.1 |
| Activated TLR2 | 1 (NS) x1.1 | 0.998 (NS) x1.1 | 1 (NS) /1 |
| Basal TLR4 | 0.754 (NS) | **0.028 (S)** | 1 (NS) |
| Activated TLR4 | 1 (NS) | **0.028 (S)** | 1 (NS) |
| Basal HLADR | 1 (NS) /1.1 | 0.185 (NS) /1.5 | 1 (NS) /1.3 |
| Activated HLADR | 0.754 (NS) /1.4 | **0.003 (S,<) /1.8** | 1 (NS) /1.3 |
| Basal CD11b | 1 (NS) /1 | 0.998 (NS) x1.1 | 1 (NS) x1.1 |
| Activated CD11b | 1 (NS) /1 | 0.998 (NS) x1.2 | 1 (NS) x1.2 |
| Basal CD35 | 1 (NS) x1 | 0.998 (NS) x1.1 | 1 (NS) x1 |
| Activated CD35 | 1 (NS) x1 | 0.482 (NS) x1.2 | 1 (NS) x1.2 |
| Basal CXCR1 | 1 (NS) /1.1 | 0.872 (NS) /1.1 | 1 (NS) x1 |
| Activated CXCR1 | 1 (NS) /1.1 | 0.998 (NS) /1.1 | 1 (NS) x1 |
| Basal CD16 | 1 (NS) /1.1 | 0.998 (NS) /1 | 1 (NS) x1 |
| Activated CD16 | 1 (NS) /1 | 0.998 (NS) x1 | 1 (NS) x1 |
| CCR2 ratio* | 1 (NS) /1.1 | 0.998 (NS) x1 | 1 (NS) x1.2 |
| TLR2 ratio* | 1 (NS) x1.1 | 0.983 (NS) x1.1 | 1 (NS) x1.1 |
| HLA_DR ratio* | 1 (NS) /1.3 | 0.983 (NS) /1.2 | 1 (NS) x1 |
| CD11b ratio* | 1 (NS) | 0.848 (NS) | 1 (NS) |
| CD35 ratio* | 1 (NS) | 0.468 (NS) | 1 (NS) |
| CD16 ratio* | 1 (NS) x1.1 | 0.983 (NS) x1.5 | 1 (NS) x1.5 |
| IL1β | 1 (NS) /1.8 | 0.983 (NS) /1.6 | 1 (NS) x1.1 |
| IL10 | 1 (NS) /1.2 | 0.998 (NS) /1.1 | 1 (NS) x1 |
| TNFα | 1 (NS) /1.6 | 0.998 (NS) /1.3 | 1 (NS) x1.3 |

**Ratio:* MFI (basal marker) /MFI (activated marker)
